# Recent advances in multimodal imaging of infections: research highlights using Nuclear-Optical imaging

**DOI:** 10.1007/s00259-025-07724-y

**Published:** 2026-01-10

**Authors:** Mick M. Welling, Cathryn H. S. Driver, Palesa C. Koatale, Tricia Naicker, Thomas Ebenhan

**Affiliations:** 1 Present Address: Leiden Preclinical Imaging Facility, Department of Radiology, Leiden University Medical Center, Leiden, 2333 ZA The Netherlands; 2 South African Nuclear Energy Corporation (Necsa), Radiochemistry, Pelindaba, 0240 South Africa; 3 Nuclear Medicine Research Infrastructure (NuMeRI), Steve Biko Academic Hospital, Pretoria, 0001 South Africa; 4 Department of Nuclear Medicine, University of Pretoria, Pretoria, 0001 South Africa; 5 Catalysis and Peptide Research Unit, University of KwaZulu-Natal, Durban, 4001 South Africa

**Keywords:** Multimodal imaging, Hybrid tracers, Infection diagnostics, Nuclear medicine (SPECT/PET), Fluorescence-Guided surgery

## Abstract

Multimodal imaging using hybrid imaging agents is a promising strategy for diagnosing and evaluating pathologies after image-guided surgical interventions. Combining optical and radioactive imaging techniques provides a comprehensive approach to monitoring and diagnosing infections, which would be more effective than routine nuclear clinical tracers for SPECT or PET imaging, thereby enabling more effective treatment as in image-guided surgery. This review summarizes the latest developments in hybrid imaging agents and vectors for radioactive and optical imaging of bacterial, fungal, and viral infections. We pinpoint the pitfalls in the current preclinical landscape for developing infection imaging tracers. Besides diagnosing and tracking pathogens, the role of optical imaging in diagnosing and aiding antimicrobial interventions, including image-guided surgery, is discussed. Finally, practical considerations are addressed for multimodal workflow challenges in preclinical infection imaging with hybrid tracers.

**Supplementary Information:**

The online version contains supplementary material available at 10.1007/s00259-025-07724-y.

## Introduction

Severe or complex infection scenarios remain a major clinical challenge, particularly in immunocompromised and post-surgical patients. In routine practice, broad-spectrum antibiotics are often administered empirically while awaiting microbiological confirmation. However, the reliable diagnosis of deep-seated infections still depends on invasive tissue biopsies followed by culturing and PCR-based identification, time-consuming procedures that are technically demanding [1]. Thus, findings may be challenging to interpret in cases of infections involving multiple strains.

The rising prevalence of multidrug-resistant organisms, driven by aging populations, increased chemotherapy use, and the growing number of orthopaedic implantations, underscores the need for more targeted antimicrobial stewardship. Broad-spectrum antibiotic overuse contributes significantly to resistance, morbidity, and mortality in hospitalized patients. To improve outcomes, clinicians desire noninvasive, pathogen-specific detection techniques that can accurately identify infection sites and monitor therapeutic responses sensitively, yet such technologies remain largely unavailable.

Nuclear imaging offers distinct advantages over conventional modalities, such as ultrasound, computed tomography (CT), magnetic resonance imaging (MRI), and microbiological culturing, including whole-body coverage, high sensitivity, and functional insight [2]. Clinically, ^99m^Tc-labelled leukocytes and ^99m^Tc- (hydroxy) methylene diphosphonate ([^99m^Tc]Tc-MDP/HDP) bone scans are widely used in single-photon emission tomography (SPECT), while 2-deoxy-2-[¹⁸F]fluoro-D-glucose ([^18^F]FDG) remains the dominant positron emission tomography (PET) tracer [3]. However, these agents primarily reflect host inflammation and lack specificity for bacterial pathogens, creating a longstanding need for novel, infection-targeted tracers [4, 5].

Recent efforts have explored PET tracers that interact with bacterial receptors or metabolic pathways and provide spatial information, namely the site(s) and extent of infection. Promising candidates include, e.g., radiolabelled ubiquicidin peptide derivatives ([^99m^Tc]Tc-/[^18^F]F-/[^68^Ga]Ga-UBI_29 − 41_), 2-deoxy-2-deoxy-2-[^18^F]fluorosorbitol ([^18^F]FDS), 6-[^18^F]fluoromaltose, [^11^C]*para*-aminobenzoic acid ([^11^C]PABA), radiolabelled D-amino acids, and radiolabelled trimethoprim analog ([^11^C]TMP, [^18^F]FPTMP) [6–9]. While many show preclinical specificity, as excellently highlighted by Ordonez and Lawal [10–12], only radiolabelled UBI_29 − 41_ [13–17] and [¹⁸F]FDS [6, 18] have progressed into various exploratory human trials [19].

Despite growing interest, the development of infection-specific tracers faces persistent hurdles, including poor or off-target pharmacology, undesired biodistribution, and limited clinical versatility [20]. These limitations hinder the ability to guide interventions such as antimicrobial therapy, debridement, and implant retention (DAIR) procedures, as well as precision surgery. Recently, optical imaging techniques have gained interest in detecting infections, complementing traditional radioactive imaging methods such as SPECT and PET [14]. Consequently, research has shifted toward multimodal (hybrid) agents that combine radioactive and fluorescent labels to support both preoperative diagnostics and intraoperative image guidance. The latter in vivo optical imaging techniques include bio- or radioluminescence, or Cerenkov luminescence [21]. Fluorescent biomarkers targeting bacterial components allow for visualization of cutaneous and subcutaneous infections with high specificity [22, 23]. Near-infrared fluorescence (NIRF) mitigates autofluorescence and improves tissue penetration [24–27], with promising candidates such as NBD- or ICG02-labelled UBI_29–41_, polymyxin-B-NBD, vancomycin-IRDye800CW, PSVue^®^794-Zn-DPA, and CNIP800 - a β-lactamase-cleavable probe for targeting of *Mycobacterium tuberculosis* [24, 25]. However, tissue attenuation and altered pharmacokinetics remain challenges [28, 29], and only a limited number of fluorophores are approved for human use [30]. There remains an unmet clinical need to better align existing surgical procedures with imaging modalities designed to improve patient outcomes [31]. To address this gap, the preclinical development of hybrid imaging agents requires innovative strategies and tailored workflows to mitigate critical pitfalls, such as non-specific uptake, suboptimal pharmacokinetics, and insufficient pathogen-targeting capabilities [6]. As in studies of hybrid tracers for image-guided surgery in oncology, imaging infections with hybrid imaging agents that can be multiplexed to confirm overlap between the radioactive and fluorescent signals. Such research is only possible if both markers are available on the same tracer. The use of a single hybrid imaging construct enables performance-guided, image-guided interventional surgery for the treatment of primary infections and metastatic clusters. This approach is critical because in orthopedic infections, even small amounts of viable, often drug-resistant bacteria that remain after the DAIR procedure may rapidly reinfect and colonize the entire prosthesis.

This review provides an overview of multimodal imaging and critically examines the limitations of current infection-targeted agents. It highlights suitable strategies, focusing on their mechanisms of action and chemical synthesis options based on currently developed multimodal probes, and exemplifies biomolecular platforms with translational potential for clinical diagnostics, surgical navigation, and therapeutic monitoring. It provides a focused discussion of the crucial alignments required for clinical decision-making, the critical gaps in bench-to-bedside translation, and practical considerations to optimise multimodal imaging workflows in preclinical infection studies.

## Multimodal imaging

A critical unmet need exists for early, pathogen-specific diagnosis to enable tailored antimicrobial therapy. The management of infectious diseases has become increasingly complex due to rising resistance among bacterial and fungal pathogens and the rapid deterioration of patient health. In such cases, surgical intervention may be necessary to excise infected tissue, and intraoperative image guidance can play a pivotal role in accurately visualizing affected areas [32]. Molecular imaging provides a powerful platform for visualizing infection dynamics in vivo, enabling real-time evaluation of therapeutic efficacy and supporting clinical decision-making with unprecedented precision [33, 34]. Targeted imaging of pathogen-specific features, such as outer membrane epitopes, biofilms, intracellular components, or metabolic activity, facilitates discrimination between infectious and non-infectious pathological processes.

Multimodal imaging (MMI), also referred to as hybrid imaging, integrates two or more complementary imaging modalities or contrast agents within a unified experimental or diagnostic workflow, yielding context-rich and anatomically detailed visualizations of biological systems. In infectious disease research, MMI is increasingly recognized as a noninvasive strategy for detecting and characterizing infections in vivo [24]. By harnessing the distinct strengths of optical and nuclear imaging, such as high sensitivity, spatial resolution, and molecular specificity, MMI enhances the detection of bacterial infections and provides insight into therapeutic responses, whether pharmacological or surgical [35].

**Fig. 1:**
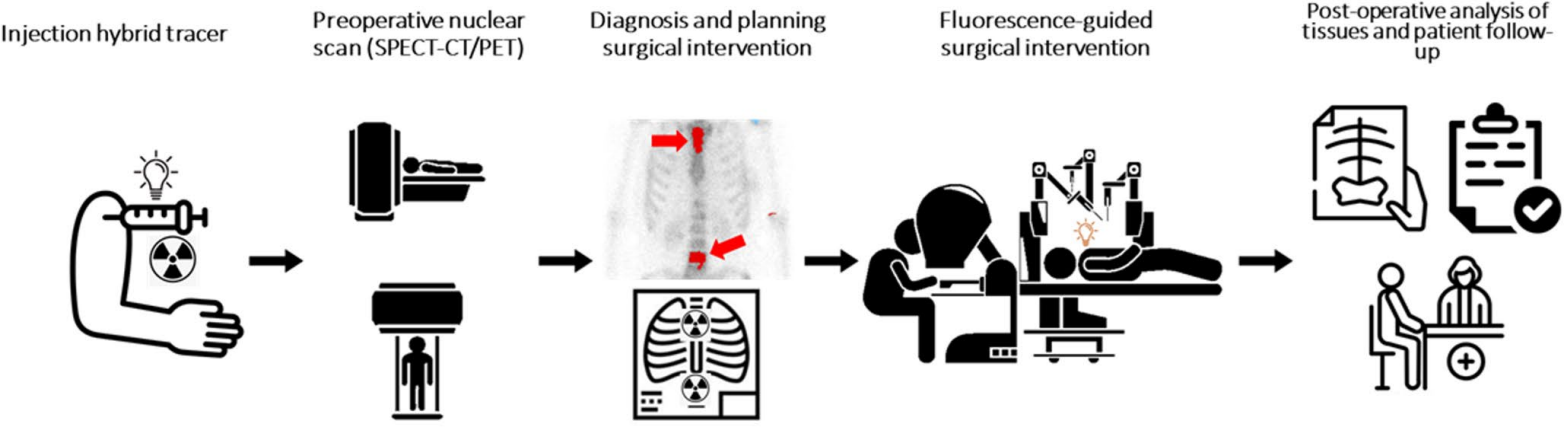
Representation of hybrid imaging and surgical intervention employing SPECT and PET imaging modalities: In nuclear imaging, after total-body imaging, primary infections and metastatic infections are located and diagnosed. Thereafter, the surgical intervention is planned to use NIRF imaging in robotic surgery or open laparoscopy. The surgeon is guided by imaging of the emitted fluorescence signal of the fluorophore accumulated in the infected lesion, which is detected by a fluorescence camera. Image created using icons from the Noun Project

MMI agents can be radiolabelled with isotopes suitable for total-body SPECT or PET imaging during the preoperative phase, enabling visualization of bacterial dissemination to affected tissues [36] (Fig. 1). These agents may also be chemically modified to incorporate fluorophores into their non-critical targeting moieties, generating dual-labelled probes for both diagnostic optical imaging and intraoperative guidance. This dual-labelling strategy, i.e., combining radioisotopes for preoperative imaging with fluorophores for optically guided surgery, has already demonstrated clinical utility in oncology [37–39]. One of the key challenges in developing such agents is selecting an optimal fluorophore that offers favourable pharmacokinetic properties [29, 40]. For optimal tissue penetration and minimal autofluorescence, emission should fall within the near-infrared window (650–850 nm), where human tissue exhibits low background signal and improved imaging contrast [41].

## Literature search and study selection

### Literature search query

The search was performed on January 20th, 2025. No starting date was applied. The PubMed/MEDLINE (NIH National Library of Medicine) and Web Of Science (WOS, Clarivate Analytics) literature databases were systematically utilized, searching for SPECT, PET, and fluorescence publications on specific imaging of infections with hybrid (radioactive and fluorescent) biomolecules only. Briefly, a tailored literature search was carried out and screened of studies present on the PubMed database first by using the following combination of keywords (“PET” OR “SPECT”) AND (infection”) AND (“optical”) AND (“fluorescence”); (“radioactive”) AND (“imaging”) AND (“infection”) AND (“optical” OR “fluorescence”). Where available, the chemical structure of the radiolabelled compounds and clinical images were shown. Additionally, the WOS, PubMed databases, and Google Search Engine were comprehensively searched for terms of interest, i.e., *“Hybrid tracers for SPECT or PET and optical imaging of infections”*,* “SPECT or PET and optical imaging of infections”*, OR *“Hybrid tracers for imaging of infections”*.

### Inclusion and exclusion criteria

One inclusion criterion was defined: papers focusing on the role of hybrid SPECT/PET/fluorescence in infection imaging (with full text available in English). Exclusion criteria were defined as follows: (***a***) papers representing research with tracers of one imaging modality, (***b***) papers out of the scope of the review, and (***c***) articles that mentioned hybrid biomarkers such as CT and MRI.

### Study selection

From the latter literature query, 78 studies were collected. The lists were screened for duplicates (which were removed), and two individuals screened the selected articles. This search strategy yielded 26 relevant publications on 15 novel biomolecules.

## MMI strategies for better imaging of infection

In the following section, we will highlight strategies that utilize MMI agents, enabling both SPECT/PET and optical imaging to study infection in vivo. These MMI agents were collected from database searches described in the previous section. Their characteristics, mechanism of action, chemical and radiochemical syntheses, combination of imaging modalities, performance, and target quantitation during preclinical infection imaging studies are reviewed.

### MMI of unique pathogenic siderophore-based iron shuttle metabolism

Invading pathogens, such as bacteria and fungi, require large amounts of iron for their virulence and survival within the host [42]. As such, these pathogens excrete secondary metabolites known as siderophores, which have a high affinity for complexing iron. In humans, iron is bound to transferrin in the bloodstream or to haemoglobin in erythrocytes. Bacteria and fungi use the siderophore system to acquire iron from their host. Bacteria and fungi subsequently reabsorb this siderophore-bound iron via specific siderophore transporters (SIT) [43]. This iron-acquisition system is highly upregulated during infection and is absent in the human body, making it specific to the pathogens.

While various pathogens can often recognize different siderophores based on structural similarities and similar transporter binding, each pathogen is known to excrete its own siderophore. Thus far, over 500 different siderophores have been identified, of which 270 have been structurally characterized [44]. Siderophores are generally classified into three main types based on their chemical structure and functional groups, namely chatecholates (including phenolates), as well as mixed types thereof. These siderophores act as hexadentate ligands that complex iron (III) (hard Lewis acid) in an octahedral geometry with very high affinity through negatively charged oxygen atoms (hard Lewis base) [45]. Based on the hexadentate coordination of the metal ion and the high affinity of oxygen donors for heavy metals, siderophores can also complex other tripositive metals such as Gallium (III), chromium (III), cobalt (III), indium (III), and zirconium (III) [46], the exact specificity of which would also be influenced by the ionic radius.

Diagnosis or therapy of the pathogen through the targeting of siderophore transporters by a siderophore with an attached diagnostic or therapeutic agent has garnered considerable research interest. For PET imaging applications of siderophores, ^68^Ga is the most favourable radionuclide since the chemistry of gallium is very similar to that of iron – equal charge and comparable radius [47, 48]. Other imaging radionuclides that have been used with siderophores include ^89^Zr and indium-111 [44].

**Fig. 2:**
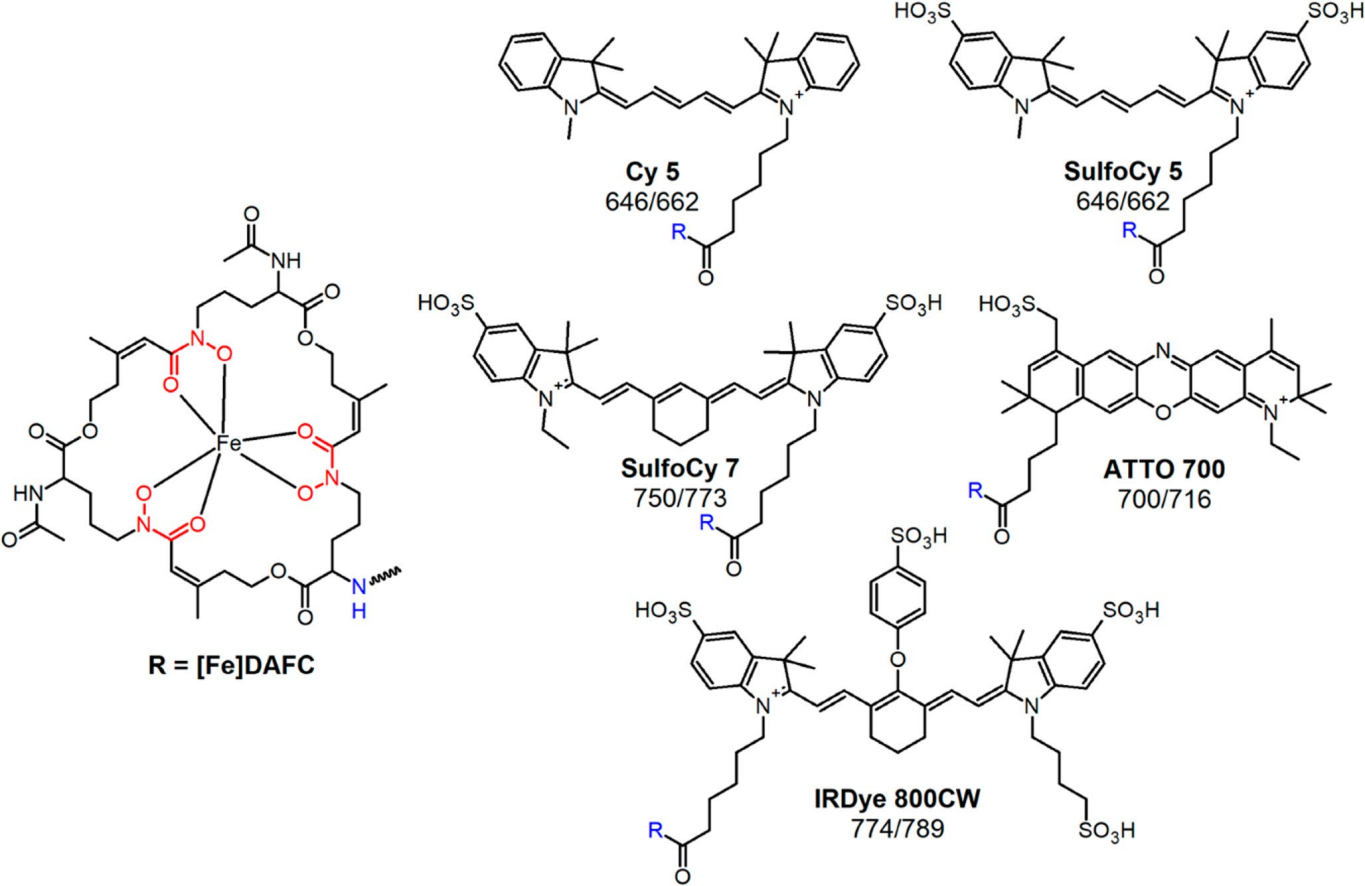
Chemical structure of diacetylfusarinine C (DAFC) and conjugated fluorophores with their corresponding absorption/emission (nm). Reproduced with permission from: [51]. Published with permission under a Creative Commons Attribution 4.0 International License. https://creativecommons.org/licenses/by/4.0/

For example, *Aspergillus fumigatus*, a human pathogen causing severe invasive fungal infections, secretes the siderophore desferri-triacetylfusarinine-C (TAFC) to acquire iron from the human host [49]. ^68^Ga-labelled TAFC was produced to enable PET imaging, with the ionic ^68^Ga(III)-TAFC complex mimicking natural iron-TAFC and entering the pathogen by the “Trojan horse” principle [46]. However, the siderophore structure will allow for hybrid labelling, i.e., while ^68^Ga (III) is complexed within, auxiliary conjugation to dyes can be considered elsewhere in the siderophore structure. TAFC is classified as a hydroxamate siderophore and has three N_2_-acetyl-N_5_-cis-anhydromevalonyl-N_5_-hydroxy-L-ornithine units ester-bonded into a cyclic system. The chemistry of these siderophores is reviewed by Al Shaer et al. [50]. In TAFC, the three amino groups are acetylated, as shown by Cunha et al. [34] that replacement of one of the acetyl groups does not alter the metal-complexation characteristics or specific recognition of the siderophore. This knowledge enables the design of siderophores functionalized with a fluorophore for optical imaging and able to complex ^68^Ga for PET imaging, forming a true MMI agent. A recent study by Pfister et al. started from ferric diacetylfusarinine C ([Fe]DAFC) and modified this compound with various fluorescent dyes to combine PET with optical imaging for hybrid applications [51] (Fig. 2).

**Fig. 3:**
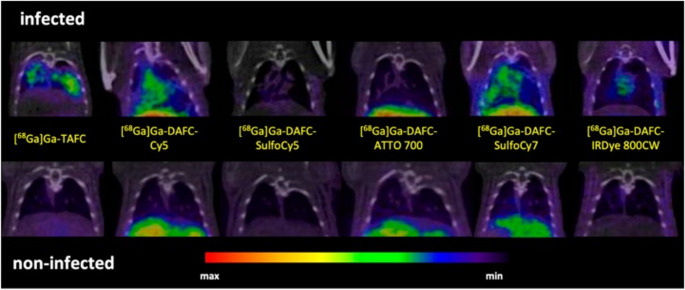
Coronal µPET/CT slices of *A. fumigatus* infected lungs (top row) and non-infected animals (bottom row) 45 min p.i. of 6 different ^68^Ga-labelled fluorophore conjugates in immunocompromised Lewis rats. The images are reproduced with permission from: [51]. Published with permission under a Creative Commons Attribution 4.0 International License.https://creativecommons.org/licenses/by/4.0/

**Fig. 4:**
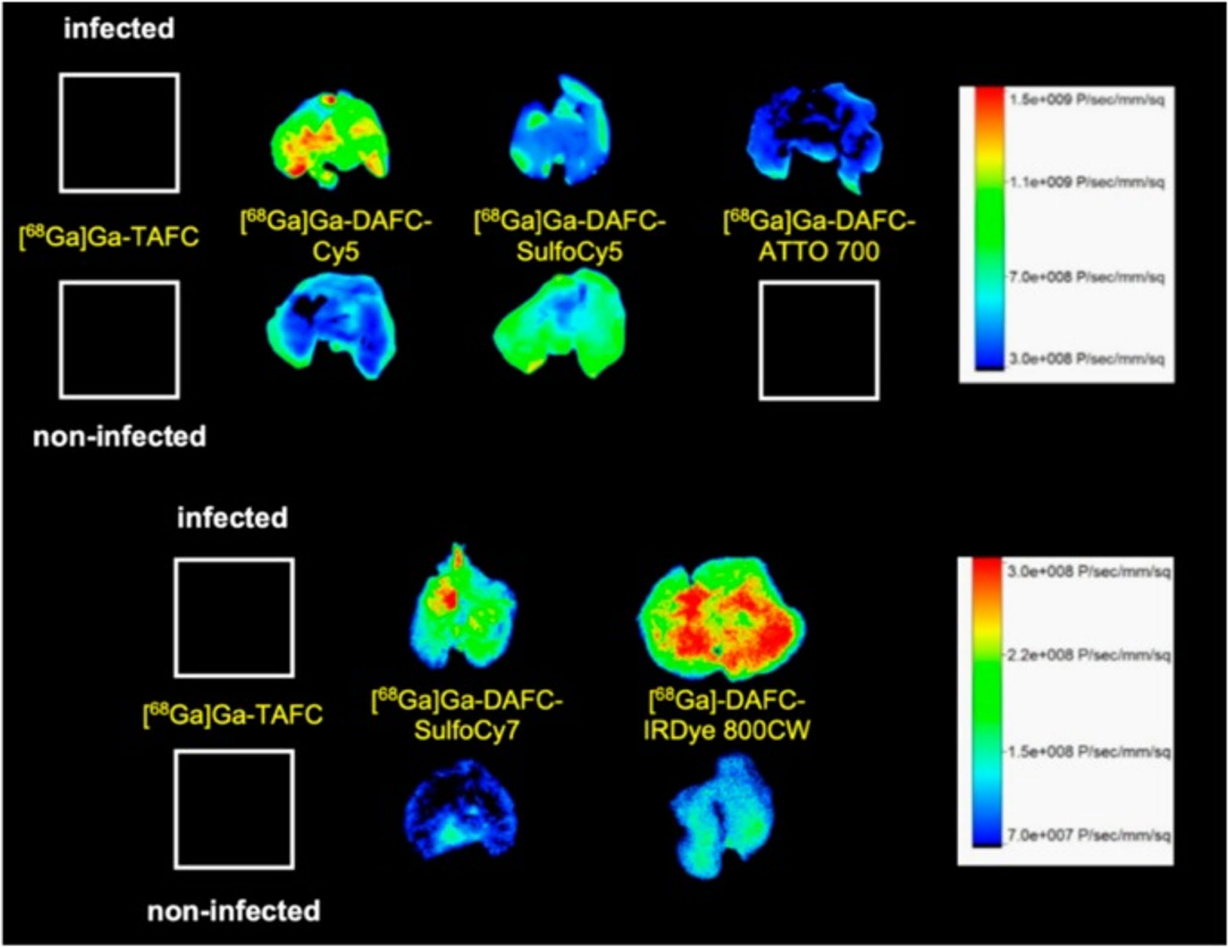
Fluorescence images of lungs excised from *A. fumigatus*-infected and non-infected Lewis rats 1.5 h after injecting different ^68^Ga-labelled fluorophore conjugates. Images reproduced with permission from: [51]. Published with permission under a Creative Commons Attribution 4.0 International License. https://creativecommons.org/licenses/by/4.0/

Briefly, five fluorescent dyes (Cy5, SulfoCy5, SulfoCy7, IRDye800CW, and ATTO700) with emission wavelengths meeting the desired 650 to 800 nm were conjugated to DAFC and subsequently labelled with ^68^Ga for in vitro and in vivo characterization as potential MMI agents. Uptake assays, growth assays, live-cell imaging, biodistribution, PET imaging, and ex vivo optical imaging of these fluorescent ^68^Ga-siderophores (control group received [^68^Ga]Ga-TAFC only) were performed in a murine lung infection model. The five hybrid conjugates were recognized by the fungal TAFC transporter MirB, and all could be used as scavengers for iron. [^68^Ga]Ga-DAFC-Cy5, [^68^Ga]Ga-DAFC-SufloCy7, and IRDye 800CW enabled the visualization of infected foci (Figs. 3 and 4).

Optical imaging of the infected lungs ex vivo corresponded well with the PET images, yielding high contrast between infected and non-infected areas. Notably, when comparing the biodistribution and infection-targeting performance of various siderophore-fluorophore compounds, even subtle differences in their chemical properties, e.g., lipophilicity, charge, and molecular weight, were found to significantly influence their pharmacokinetics and ability to accumulate within bacterial pathogens. Still, the authors expect that the concept of this MMI targeting strategy will also be applicable to other siderophore structures.

### MMI based on bacterial carbohydrate metabolic activity

Bacteria require carbohydrates as a source of energy and ingest maltodextrins through the specific maltodextrin transporters [52]. Maltodextrins are glucose polymers with repeating D-glucose units linked through 1,4-α-glycosidic bonds. Maltodextrins are digested in blood serum through starch-degrading enzymes, which recognize either the reducing end of the maltodextrin with the anomeric centre (degraded by α-amylase) or the non-reducing end (degraded by α-glucosidases) [53]. These terminal monosaccharides are also important for recognition by the maltodextrin transporters. The outer membrane transporter (LamB) in Gram-negative bacteria recognizes the non-reducing end of the maltodextrin and transports the polysaccharide into the periplasm. In this space, the maltodextrin is further recognized by the maltodextrin-binding protein (MBP) or the maltose-binding protein (MalE) and taken to the inner membrane transporter (MalFGK_2_), which binds the terminal glucose with the free anomeric hydroxyl group to take it into the cytoplasm. Gram-positive bacteria, lacking an outer membrane, are proposed to possess a maltodextrin transporter analogous to MalFGK2, which requires a free reducing end of the polysaccharide [54]. These characteristics of maltodextrin are important when designing a bacterial targeting agent for the maltodextrin transporters, as the bacterial uptake will be affected. It was noted, however, that functionalization of the anomeric position was more tolerated than functionalization of the non-reducing terminus, which excludes recognition by LamB and MBP and therefore negates any compound uptake [51, 55].

**Fig. 5:**
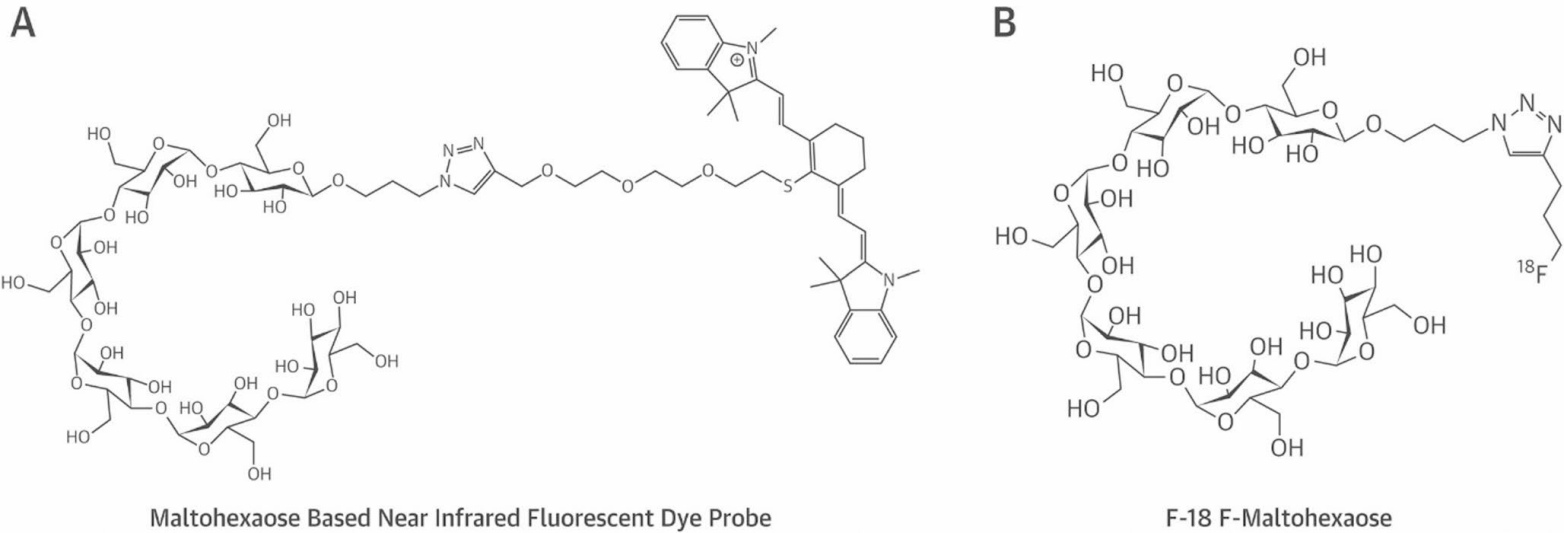
**A** Chemical structure of maltohexaose-based near-infrared fluorescent dye probe and **B** [^18^F]fluoromaltohexaose. Image reproduced with permission from Takemiya et al. [56]. Published with permission under Creative Commons Public Domain Mark 1.0. https://pmc.ncbi.nlm.nih.gov/about/copyright/

Maltodextrins have been explored as MMI agents for bacterial infection imaging, with several maltohexaose-based probes showing promising results [56]. Takemiya et al. demonstrated that both fluorescent and radiolabelled maltohexaose derivatives could detect infections associated with cardiac implants. These compounds were conjugated either to the near-infrared dye IR-786 (excitation/emission: 710/790 nm) via a triazole-PEG linker, or to a [¹⁸F]-fluorinated propyl triazole linker at the anomeric position of the terminal sugar (Fig. 5A–B).

**Fig. 6:**
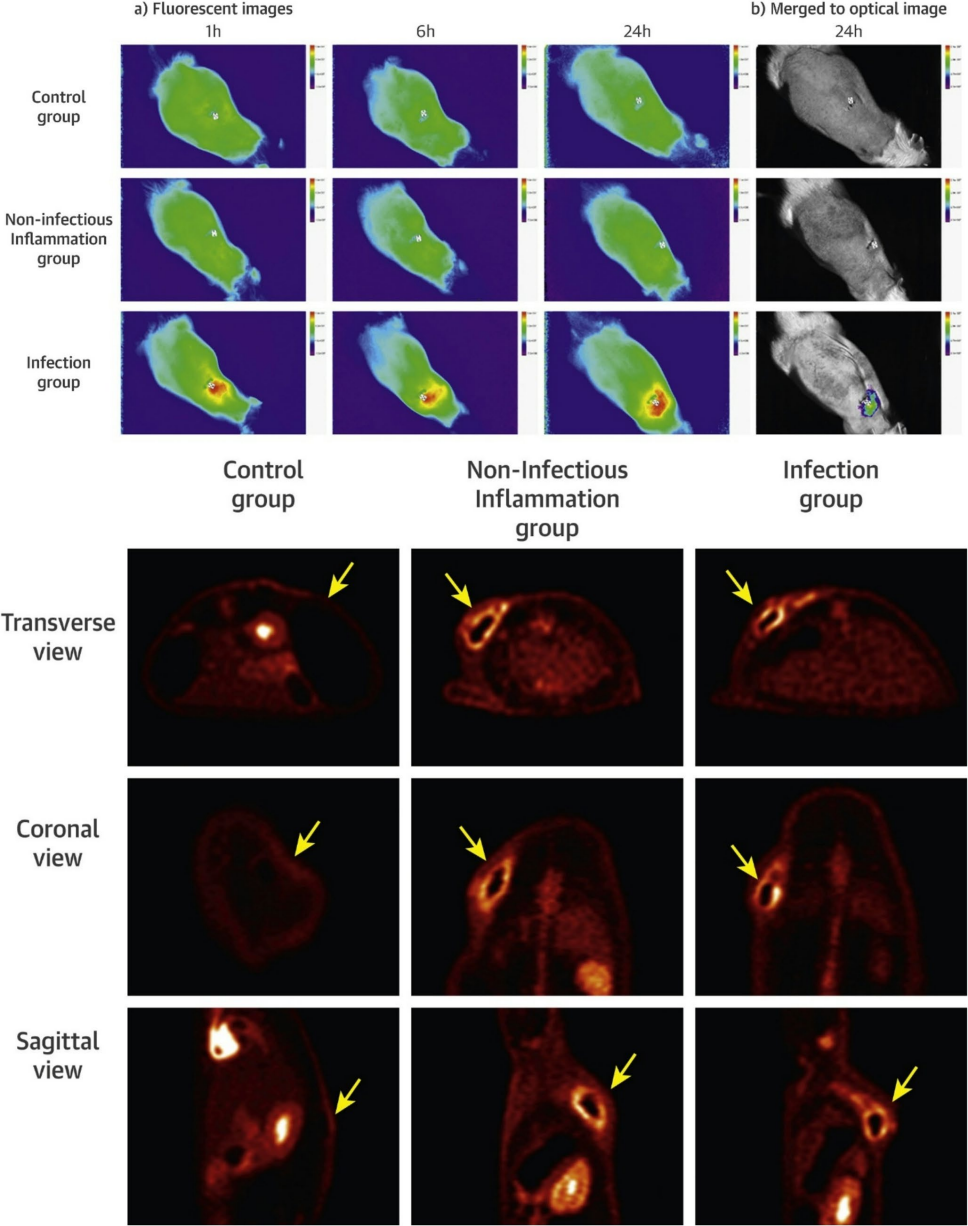
(*Top panel*) Accumulation of the maltohexaose fluorescent dye probe was only observed in the infected tissue from 1 h after the injection. (*Bottom panel*) With PET imaging, the accumulation of [^18^F]fluoromaltohexaose was most prominent in the infection and the inflammation groups. Yellow arrows indicate the location of the area of interest in all groups, including the control group. Image reproduced with permission from Takemiya et al. [56]. Published with permission under Creative Commons Public Domain Mark 4.0. https://pmc.ncbi.nlm.nih.gov/about/copyright

The resulting probes, i.e., maltohexaose dye probe (MDP) and [¹⁸F]fluoromaltohexaose, were evaluated in a cardiac device infection model, where they demonstrated specificity and sensitivity (Fig. 6). The results indicated that maltohexaose-based imaging probes were average in specificity but high in sensitivity for diagnosing infections associated with implantable cardiac devices [56]. However, the study’s design required the co-injection of two separately labelled probes, which limited its utility as a true MMI agent. Additional concerns include uncertainty over bacterial uptake, i.e., whether the probes are internalized or merely bind to the membrane, given that maltodextrin transport via MalFGK₂ requires a free anomeric centre. Furthermore, probe stability remains questionable, as maltodextrins with non-reducing ends are susceptible to enzymatic degradation in serum. Similar, complex, pathogen-unique sugars may be recommended for exploitation in their development as a potential MMI agent.

### MMI of viral infections by targeting translocator protein activity

Advances in fluorescence imaging have enabled the identification of biomolecules involved in viral-host interactions for several clinically significant viruses, including HIV, hepatitis C, and Ebola [57, 58]. The emergence of SARS-CoV-2 in 2019 created an urgent need for rapid, noninvasive diagnostic imaging tools, including SPECT and PET. One approach involved targeting the translocator protein (TSPO), an 18 kDa mitochondrial membrane protein that is upregulated in activated macrophages and phagocytic cells, making it a valuable marker for imaging pulmonary inflammation and infection. The small, pyrazolopyrimidine-based ligand DPA-713 exhibits a high binding affinity (Ki = 4.7 nM) to TSPO and has successfully been radiolabelled with ^11^C, ^18^F, ^124^I, and ^125^I, and fluorescently tagged with IRDye680LT (excitation/emission: 676/693 nm) or IRDye800CW (excitation/emission: 774/789 nm) [59]. Diagnostic labelling has been achieved either through electrophilic radiohalogenation of aromatic rings or functionalization of the phenolic hydroxyl group.

**Fig. 7:**
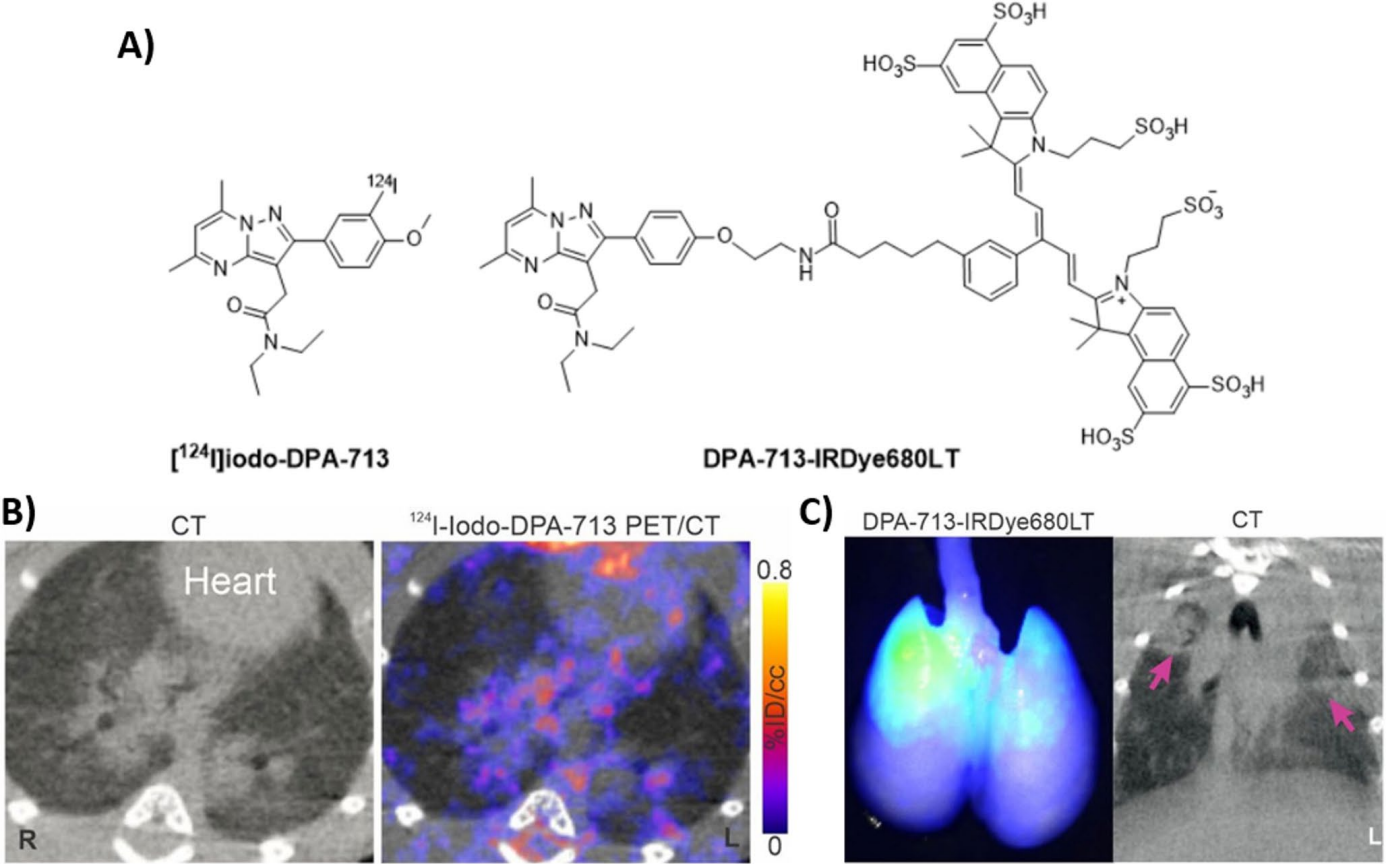
**A **Structure of [^124^I]*iodo*-DPA-713 and DPA-713-IRDye680LT; SARS-CoV-2 related pulmonary inflammatory changes visualized by **B** CT and [^124^I]*iodo*-DPA-713 PET/CT, imaging; and **C** CT and DPA-713-IRDye680LT optical imaging. Image adapted from Ruiz-Bedoya et al. [60]. Published with permission, Copyright World Molecular Imaging Society

To visualize SARS-CoV-2 invasion in the lungs of infected hamsters, researchers co-administered [¹²⁴I]*iodo*-DPA-713 and its fluorescent analog DPA-713-IRDye680LT (Fig. 7A) [60].

In the context of viral infection imaging, both [¹²⁴I]*iodo*-DPA-713 and DPA-713-IRDye680LT were able to visualize pneumonic changes associated with SARS-CoV-2 infection in preclinical models (Fig. 7B). Despite notable differences in molecular weight (489.36 vs. 1572.84 g/mol) and lipophilicity, both compounds demonstrated effective TSPO targeting, suggesting that these physicochemical disparities did not critically impair tracer performance. While this study (at the time) provided a proof of concept for the application of specific noninvasive biomarkers to image SARS-CoV-2-associated pulmonary inflammation, it was, however, limited by the need to co-inject two separate agents, i.e., one radiolabelled and one fluorescent, which complicates the workflow and may hinder translational scalability. More importantly, the lack of direct viral targeting and the limited specificity of TSPO for SARS-CoV-2-infected cells raise concerns about the clinical utility of DPA-713 as a diagnostic tool for viral infections. While imaging activated host immune cells (e.g., macrophage migration) remains a valid strategy for assessing inflammatory responses, TSPO expression is not exclusive to infection. It may be elevated in a range of non-infectious conditions, including trauma, neurodegeneration, and sterile inflammation [61]. Therefore, future research should consider alternative cellular targets, such as metabolic signatures or immune checkpoints, that may offer greater specificity and functional relevance for the development of next-generation MMI agents.

### MMI of infections using bacteria‑specific antibody

Antibody-based imaging enables the noninvasive detection of infection by targeting pathogen-specific antigens or immune markers, thereby helping to distinguish septic from sterile inflammation and guide image-based interventions [62]. In 2019, a hybrid imaging agent was developed using the fully human monoclonal antibody 1D9, which specifically targets the immunodominant staphylococcal antigen A (IsaA) expressed on the surface of *S. aureus*. IsaA is involved in bacterial cell wall remodelling and biofilm formation during infection, making it a suitable target for antibody-based imaging. This antibody was dually labelled with the positron-emitting radionuclide ⁸⁹Zr for PET imaging and the near-infrared fluorophore NIR680 (excitation/emission: 680/715 nm) for optical visualization. Radiolabelling with the ⁸⁹Zr radionuclide was achieved via chelation to a deferoxamine (DFO) moiety conjugated to the antibody, a well-established method for stable zirconium coordination. The NIR680 fluorophore was covalently attached through amine-reactive NHS ester chemistry, allowing dual-modality imaging without compromising antibody binding affinity. The resulting tracer, [⁸⁹Zr]Zr-NIR680-1D9, was evaluated in a complex (i.e., more translatable) murine spinal implant infection model to differentiate septic from aseptic inflammation using PET/CT and in vivo fluorescence imaging [63].

**Fig. 8:**
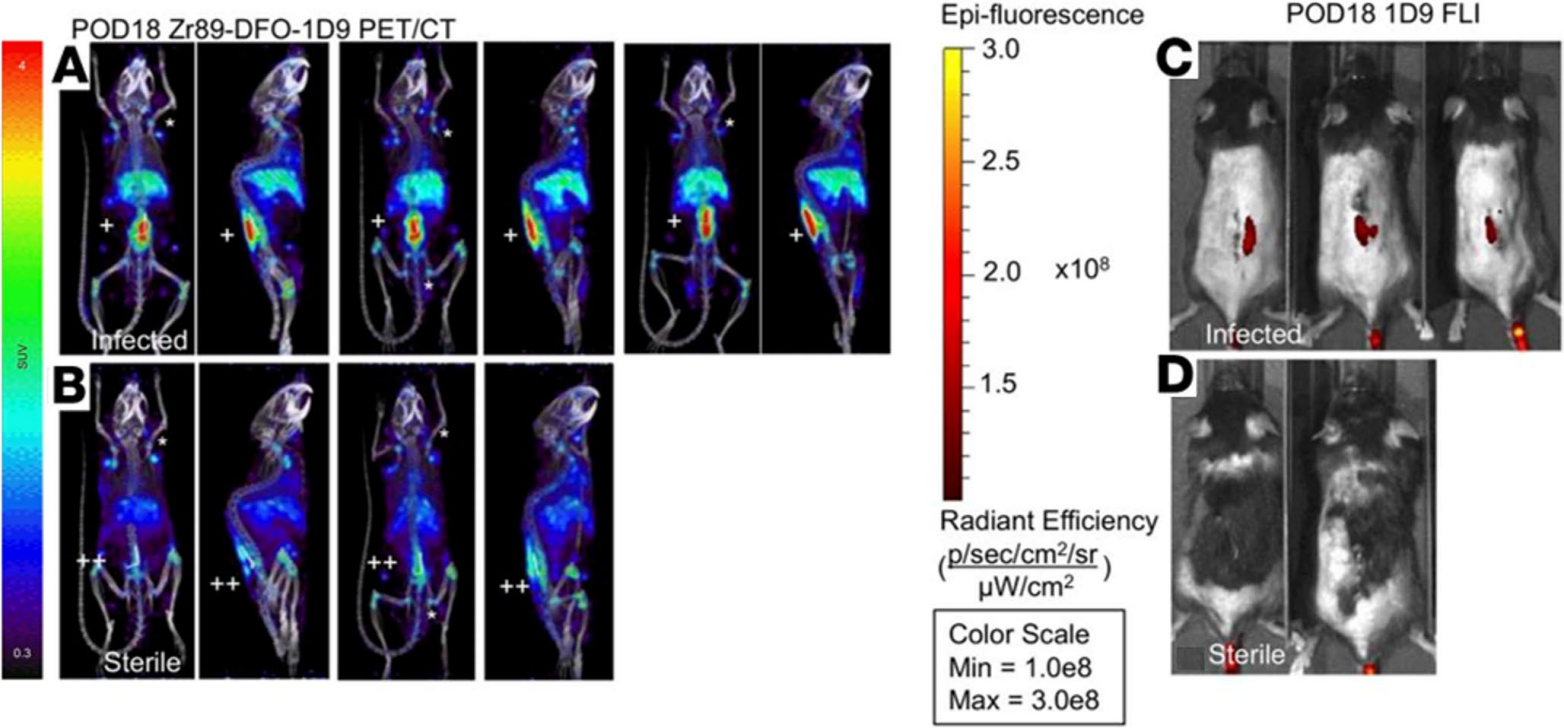
Proof of concept for [^89^Zr]Zr-NIR680-1D9 MMI. [^89^Zr]Zr-NIR680-1D9 PET/CT imaging was performed on POD 18 (chronic infection) in **A** infected mice and **B** sterile mice. **A** representative coronal and sagittal PET images of 4 infected mice, and **B **selected coronal and sagittal PET images of 2 sterile mice. Fluorescence images of **C** infected or **D** sterile mice. Adapted from Zoller et al. [63]. Published with Permission, Copyright American Society for Clinical Investigation.https://insight.jci.org/kiosks/terms

**Fig. 9:**
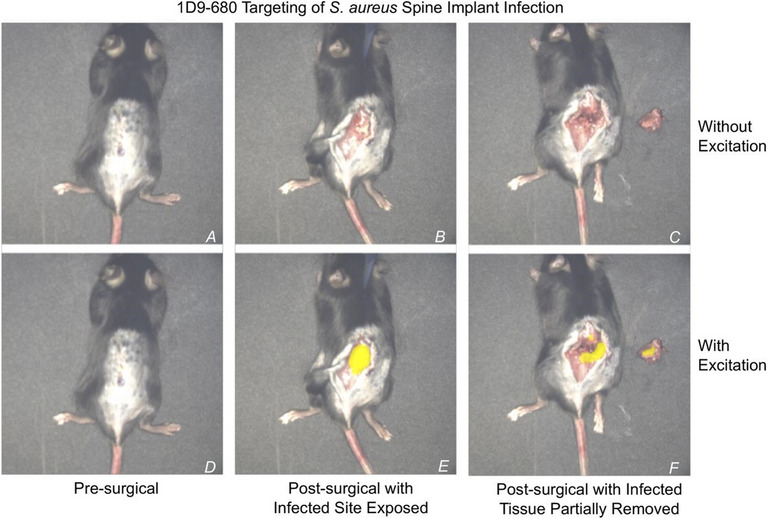
NIR680-1D9 targeting of *S. aureus* in spine implant infections. Following proof of infection, implant infection was visualized by [^89^Zr]NIR680-1D9. On day 10, 48 h after the antibody probe was administered intravenously. injection 1D9 on day 8, a mouse with an acute spinal implant infection was isolated under the image-guided surgery system and analysed both with and without fluorescent excitation at 680 nm. No fluorescent signal was visible before skin incisions **A** and **D**. Following skin incisions **B** and **E**, a fluorescent signal was only present with excitation. Following partial excision of infected tissue, as identified by fluorescent signals **C** and **F**, the fluorescent signal remained present both in the host and explanted tissue. Image adapted from: [63]. Published with Permission, Copyright American Society for Clinical Investigation.https://insight.jci.org/kiosks/terms

In this study, [⁸⁹Zr]Zr-NIR680-1D9 demonstrated high specificity for *S. aureus*, enabling precise localization of infected tissue (Figs. 8 and 9), supporting its potential to improve the clinical management of implant-associated infections. However, the tracer’s specificity is limited to IsaA-expressing *S. aureus* strains, and the long circulatory half-life of the full-length antibody (> 3 days) contributes to elevated background signal. To address this, alternative formats such as antibody fragments, affibodies, or engineered minibodies have been proposed to reduce systemic retention while maintaining target affinity [5, 64].

### MMI of electrostatic targeting of Lipopolysaccharide-Rich bacterial membranes

Prokaryotic membranes represent a multifaceted target for MMI, offering access to pathogen-specific surface epitopes, metabolic transporters, and structural motifs that distinguish infectious agents from host tissue. Their unique lipid composition, dynamic protein expression, and accessibility in vivo enable the development of hybrid imaging agents for precise detection, characterization, and intraoperative visualization of bacterial and fungal infections. This strategy aligns with emerging efforts to improve infectious disease imaging through molecularly targeted probes that exploit microbial membrane features [65]. Positively charged zinc(II)-dipicolylamine (ZnDPA) analogs electrostatically interact with negatively charged lipopolysaccharide (LPS) residues on the outer membrane of bacterial surfaces and the outer membrane of parasites [66]. These coordination complexes exploit the polyanionic nature of LPS, particularly its phosphate-rich domains. The ZnDPA moiety hereby forms stable complexes with phosphate groups via chelation, while its overall cationic charge facilitates membrane association through ionic attraction. This dual mechanism, i.e., phosphate coordination and electrostatic binding, enables ZnDPA-based molecules to accumulate at infection sites with high specificity. For example, fluorescently labelled ZnDPA compounds have demonstrated rapid uptake into parasite cytosol and selective toxicity against *L. major promastigotes*, with minimal off-target effects on mammalian cells [67, 68]. Additionally, radiometric fluorescent sensors incorporating ZnDPA have shown sensitive detection of LPS in aqueous environments, confirming their utility in both diagnostic imaging and environmental monitoring [69]. This targeting strategy was also evaluated for imaging bacterial infections using either radioactive or fluorescent techniques [67, 70–73].

**Fig. 10:**
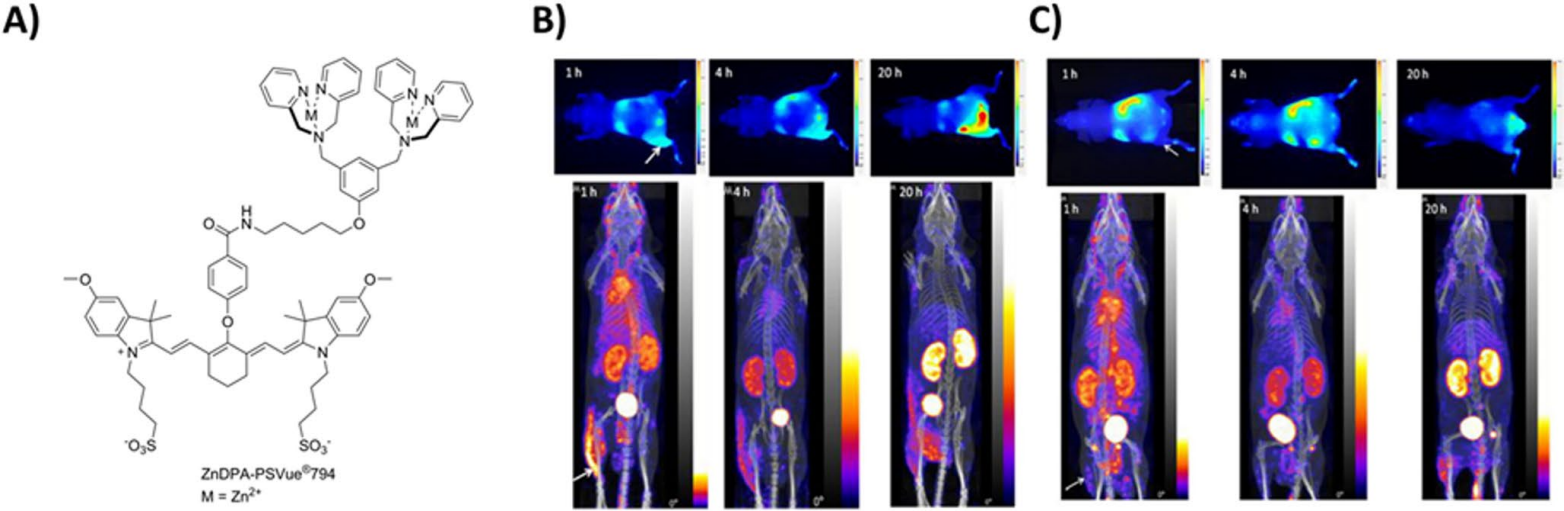
Proof of concept for MMI of lipopolysaccharide-rich bacterial membranes **A** chemical structure of PSVue^®^794-conjugated ZnDPA. Optical images (top panels) and projections of PECT/CT acquisitions (bottom panels) of **B** a *S. pyogenes*-infected mouse and **C** LPS-induced inflammation mouse at 1, 4, and 20 h post-administration of a cocktail consisting of DPA-PSVue^®^794 and [^111^In]In-DOTA-ZnDPA. The arrows indicate the sites of infection and inflammation. Adapted from Liu et al. [74]. Published with Permission, Copyright Elsevier Inc

Another ZnDPA-based MMI approach utilized an indium-111 radiolabelled compound, [¹¹¹In]In-DOTA-_(biotin/SA/biotin)_-ZnDPA, in which biotinylated ZnDPA and DOTA-biotin were linked via streptavidin (SA) (Fig. 10A). ^111^In was chelated by the DOTA-biotin moiety for SPECT imaging and ex vivo biodistribution studies. At the same time, ZnDPA enabled selective targeting of negatively charged bacterial membranes [74]. Although a true hybrid tracer was not used, co-injection with a fluorophore-conjugated ZnDPA analog (PSVue^®^794; excitation/emission: 787/808nm) allowed dual SPECT and optical imaging. Fluorescence and SPECT imaging (Fig. 10B-C) showed substantial accumulation of tracer in *S. pyogenes*-infected thigh muscle at 1-, 4-, and 22-hours post-injection. At 22 h, the infection-to-normal muscle ratio averaged at 2.8, significantly higher (*p* < 0.01) than the inflammation-to-normal muscle ratio of 1.0 for LPS-induced sterile inflammation. This combined approach successfully distinguished bacterial infections from LPS-induced sterile inflammation and demonstrated the potential for structural optimization toward clinical translation.

A key limitation of positively charged ZnDPA is its non-specific binding to apoptotic and necrotic mammalian cell membranes, making it a marker of infection-associated tissue damage rather than solely pathogen-specific uptake [68]. Rice et al. concluded that for ZnDPA, fluorescence and nuclear imaging applications include the detection of diseases such as cancer, neurodegeneration, arthritis, and microbial infections, as well as the quantification of therapy-induced cell death.

### MMI using a radiolabelled Vancomycin derivative

**Fig. 11:**
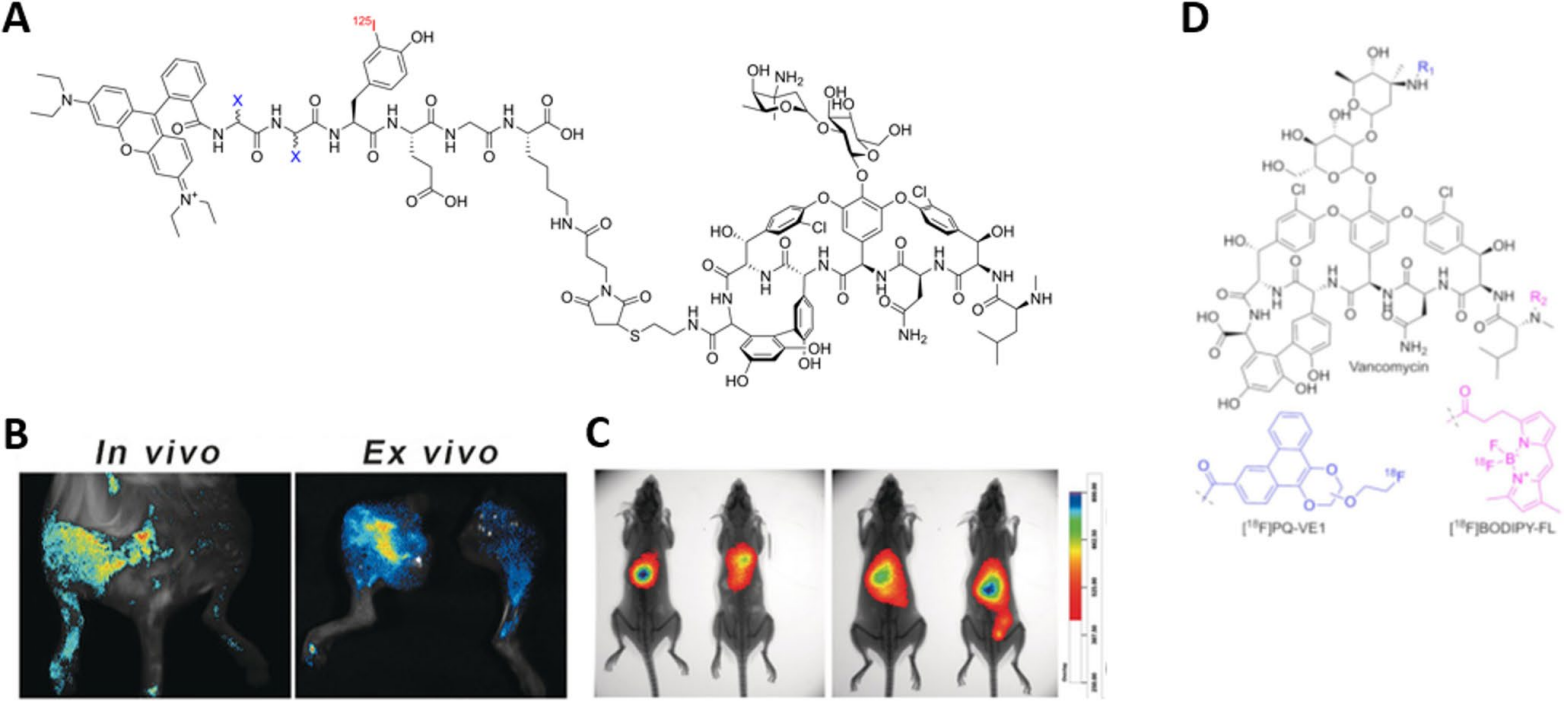
Development of hybrid vancomycin derivatives for imaging of resistant bacteria. **A** Chemical structure of [^125^I]*iodo*-Rho‐Vancomycin (X = CH2Ph). **B** In vivo and ex vivo fluorescence imaging of an infected mouse with MRSA‐induced myositis (left hind leg) and *E. coli*‐induced myositis (right hind leg) 2 h after i.v. injection. **C** In vivo SPECT imaging with [^125^I]*iodo*‐Rho‐Vancomycin in mice with MRSA-derived pneumonia (overlay of X‐ray and isotope signals). Adapted from Yang et al. [77]. Published with permission by John Wiley and Sons, **D **Chemical structures of vancomycin, [^18^F]PQ-VE1, [^18^F]FB, and [^18^F]BODIPY-FL. The nucleophilic amine moieties in vancomycin are designated as R1 and R2 for the primary and secondary amines, respectively, as adapted from Spoelstra et al. [78]. Published with permission under a Creative Commons Attribution 4.0 International License. https://creativecommons.org/licenses/by/4.0/

As highlighted by Gouws et al. in 2022, antibacterial drugs can serve as compelling dual-purpose platforms for MMI, owing to their well-characterized mechanisms of action and the extensive chemical knowledge available for strategic functionalization [75]. Vancomycin is a glycopeptide antibiotic with a cup-like structure that attaches to the D-Ala-D-Ala terminal of the peptidoglycan pentapeptide in Gram-positive bacteria through five hydrogen bonds. These bonds allow vancomycin to also function as an affinity ligand (functioning on its free carboxylic acid or amine groups), facilitating the accumulation of dyes on bacterial cell walls. The use of fluorescein-labelled vancomycin for imaging bacterial infections is, however, limited by the inability to detect deep-tissue infections because of the scattering and absorption of photons by tissue [76]. To overcome these issues, a hybrid vancomycin-based radiopharmaceutical was introduced that contained the fluorescent dye rhodamine B (introduced via NHS ester chemistry to attach to lysine residues or hydroxyl groups on vancomycin) as well as the radioisotope Iodine-125 (i.e., creating [^125^I]*iodo*-Rho-vancomycin through electrophilic substitution on aromatic rings) (Fig. 11A) [77]. This design also leveraged vancomycin’s in situ self-assembly properties to form nanoaggregates upon binding to bacteria. Mice were infected in the right thigh muscle with methicillin-resistant *S. aureus* (MRSA), whereas the contralateral thigh muscle was infected with *E. coli*, reflecting the bias in labelling Gram-positive bacteria. Within 2 h after injection using SPECT, [^125^I]*iodo*-Rho-vancomycin (excitation/emission: 540/565nm) showed an 8.7-fold higher accumulation in MRSA-infected thigh muscles than in muscles infected with *E. coli*. (Fig. 11B). Fluorescence imaging yielded a 3.9-fold increase in uptake in MRSA-infected thigh muscles with [^125^I]*iodo*-Rho-vancomycin compared to control tissue (Fig. 11C). SPECT imaging of pulmonary infections by MRSA with [^125^I*]iodo*-Rho-vancomycin yielded about 8.9- to 13.3-fold higher lung-to-background ratios than a control radiopharmaceutical (a non-cell-binding variant of [^125^I]*iodo*-Rho-vancomycin). This study underlines that [^125^I]*iodo*-Rho-vancomycin selectively accumulates on the bacterial membranes of Gram-positive bacteria. However, its dependence on self-assembly for signal enhancement may lead to variability influenced by bacterial surface conditions and environmental factors. While promising, further research is needed to validate the probe’s toxicity and effectiveness across diverse bacterial strains, as well as to evaluate its performance in clinical settings. More recently, an ^18^F-labelled PET tracer based on vancomycin and BODIPY (excitation/emission: 504/511nm) was developed (Fig. 11D); however, no convincing results on bacterial imaging have been available to date [78, 79]. Some shortcomings relate to the agent itself, which was expected to selectively bind Gram-positive bacteria, also accumulated within *E. coli*-infected tissue. This phenomenon was not observed in vitro, suggesting an unknown in vivo mechanism that may affect the specificity. Additionally, the immune response or inflammation levels may have influenced tracer accumulation, affecting the specificity and reliability of bacterial detection.

Leveraging vancomycin’s well-characterized mechanism of action to develop hybrid imaging agents is a promising strategy for bacterial imaging, particularly for selective targeting of Gram-positive bacterial surfaces. This dual-functional construct enables both deep-tissue nuclear imaging and surface-level fluorescence visualization, facilitating preoperative diagnosis and intraoperative guidance. However, as with many antibiotic-based imaging agents, a key limitation remains: the reduced efficacy and specificity in the context of vancomycin-resistant bacterial strains [80, 81]. Resistance mechanisms, such as alterations to the D-Ala-D-Ala binding site in peptidoglycan precursors, significantly reduce vancomycin’s affinity, potentially leading to compromised imaging results. This limitation underscores the need for alternative or complementary targeting strategies, such as modified tracers that bind to resistance-associated bacterial markers, or combination imaging agents to improve diagnostic accuracy in resistant infections.

### MMI of bacteria using an antimicrobial peptide

**Fig. 12:**
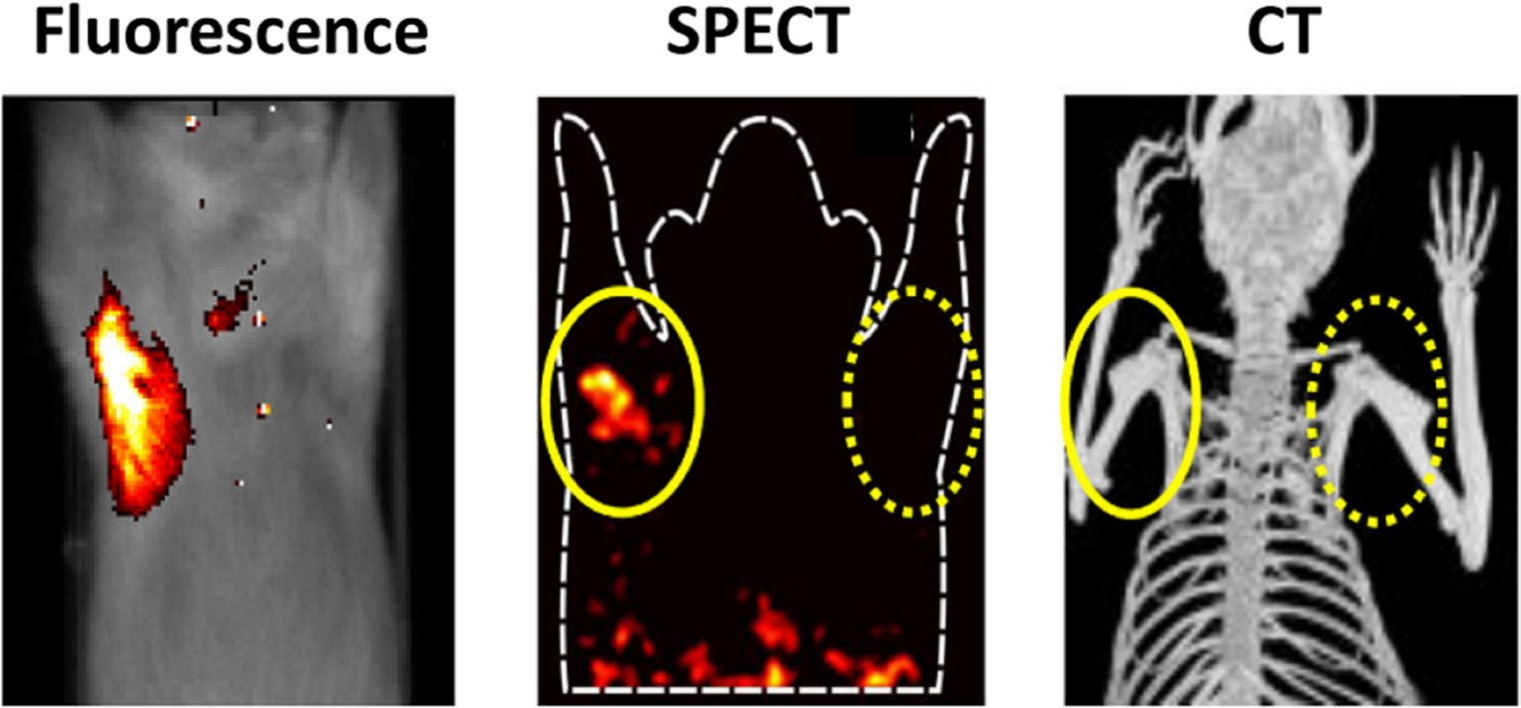
MMI uses an antimicrobial peptide. Typical hybrid in vivo imaging of *S. aureus* infections with [^111^In]In-DTPA-Cy5-UBI_29–41_ in the paw muscle in mice at 2 h after tracer injection. In the anterior images, infected muscles are indicated with oval shapes, and the contralateral non-infected muscles are indicated with dotted oval shapes. Adapted from: [89]. Published with permission, Copyright 2015, American Chemical Society

Antimicrobial peptides (AMPs) are studied for their effectiveness in eliminating a broad spectrum of pathogens, including Gram-positive and Gram-negative bacteria, fungi, parasites, and viruses [82]. Cationic AMPs bind to negatively charged lipoteichoic acid, phospholipids, and lipopolysaccharides on bacterial membranes [83, 84]. In low quantities, i.e., in tracer doses, radiolabelled antimicrobial peptides have successfully imaged pathogens in infections [85, 86]. After its introduction in 2000 [87], a radiolabelled AMP of ubiquicidin (UBI), [^99m^Tc]Tc-UBI_29 − 41_ (UBI_29 − 41_: Thr-Gly-Arg-Ala-Lys-Arg-Arg-Met-Gln-Tyr-Asn-Arg-Arg), has been the most prominent candidate for specific imaging of infections, both in preclinical studies and patients. UBI is a naturally occurring, cation-rich AMP found in human neutrophils and endothelial cells, serving as a first line of defence. About 70 preclinical studies have been conducted in various experimental models using radiolabelled ([^99m^Tc], [^111^In], [^123^/125I], [^67/68^Ga], [^18^F]) and fluorescent-labelled (ICG02, NBD, Cy5, or Cy5.5) agents. UBI_29 − 41_ has demonstrated value in targeting pathogens in vitro and in vivo [10, 68] as well as in monitoring the effects of antimicrobial drugs [88]. The translation of UBI_29 − 41_ into a kit formulation for radiolabelled UBI_29 − 41_ has yielded approximately 45 published studies in various clinical infection settings (FUO, osteomyelitis, pneumonia, endocarditis, and prosthetic infections) for SPECT and PET imaging (Fig. SI-1A). Almost a decade ago, [^111^In]In-DTPA-Cy5-UBI_29 − 41_ (Fig. SI-1D) was developed for hybrid SPECT and fluorescent imaging of bacterial infections in a preclinical model of inoculated bacteria in mice [89]. For MMI, a dual-labelled [^99m^Tc]Tc-UBI_29 − 41_-Cy5 construct (excitation/emission: 649/666nm) allowed radioactive and fluorescence imaging in experimental infections in mice (Fig. 12). Radioactivity-based measurements yielded infection-to-muscle ratios of 2.82 ± 0.32 for *S. aureus* and 2.37 ± 0.05 for *K. pneumoniae*, while fluorescence-based measurements of the same compound yielded ratios of 2.38 ± 0.09 and 3.55 ± 0.31, respectively. As with many other antimicrobial peptides, UBI_29 − 41_ is generic, meaning that it will associate with all types of bacteria (and other pathogens), but UBI_29 − 41_-based tracers cannot differentiate between various strains of bacteria.

More recently, a [^99m^Tc]Tc-UBI_29 − 41_-Cy5-N_3_ azide-functionalized variant was generated (Fig. SI-1B) to allow multimodal tracking of *S. aureus* infections in mice [90]. The role of the azide is explained below. The tracer contains a fluorescent and a radioactive moiety that could be used for [^99m^Tc]Tc-UBI_29 − 41_-Cy5-labelled bacteria to study in vivo bacterial tracking. Longitudinal imaging of a thigh-muscle infection with *S. aureus* was performed. Mice carrying a bacterial infection were imaged using microSPECT and a fluorescence-imaging 2D modality at various intervals over 28 h. SPECT and fluorescence imaging in mice demonstrated rapid retention of the radiolabelled bacteria following inoculation in the thigh muscle. Together with bacterial replication in the infected tissue, the signal intensity in the abscess decreased within a 28 h time frame: 52% of the total injected radioactivity per gram of tissue (%ID/g) at 4 h after infection vs. 44%ID/g at 28 h (15% decrease). After inoculation, only a small share of the bacteria disseminated from the inoculation site, and *S. aureus* cultures could be obtained in radioactive urine samples. Thus, imaging of the bacterial tissue burden with [^99m^Tc]Tc-UBI_29 − 41_-Cy5 allowed noninvasive bacterial-cell tracking for 28 h. For extended imaging, the [^111^ln]In-DTPA-Cy5-UBI_29–41_ can prolong the monitoring window to at least one week. Given the versatility of the presented bacterial-tracking methods, this concept allows for precise imaging capabilities during so-called “controlled-human-infection” studies.

Additionally, a follow-up study was performed to use the azide function of [^99m^Tc]Tc-UBI_29 − 41_-Cy5-N_3_ to explore the possibility and evaluation of “click” chemistry on invading bacteria in mice [91] (Fig. SI-1D). Functionalization of the UBI peptide with azide groups has been employed in pretargeting strategies for infection imaging. Tissue pretargeting is a validated approach for in vivo delivery of diagnostic or therapeutic payloads. As demonstrated by the aforementioned studies and others, the UBI scaffold enables the selective accumulation of bacteria at specific sites. The pretargeting concept can be realized through various conjugation strategies, including bioorthogonal click chemistry and modular linker systems, allowing flexible integration of nuclear and optical imaging modalities. One such strategy is based on copper-free “click” chemistry. These reactions have shown in vivo potential for imaging and radionuclide therapy. Still, this conjugation strategy has not yet been explored in combination with tracking and tracing of single-cell organisms. The study with [^99m^Tc]Tc-UBI_29 − 41_-Cy5-N_3_ evaluated the in vivo efficacy of strain-promoted azide-alkyne cycloaddition reactions to achieve imaging and pretargeting of azide-functionalized *S. aureus*. Secondary targeting was realized following the intravenous administration of ^111^In-labelled diethylenetriaminepentaacetic acid-dibenzocyclooctyne ([^111^In]In-DTPA-DBCO). To assess the click reaction efficiency in vivo, the biodistribution of ^99m^Tc- and ^111^In was measured using SPECT image-derived concentration and ex vivo tissue uptake (% ID/g). Ex vivo confocal fluorescence imaging was carried out on excised tissue samples to confirm the presence of functionalized bacteria. In vitro data confirmed the effectiveness of click reactions on the bacterial membrane.

**Fig. 13:**
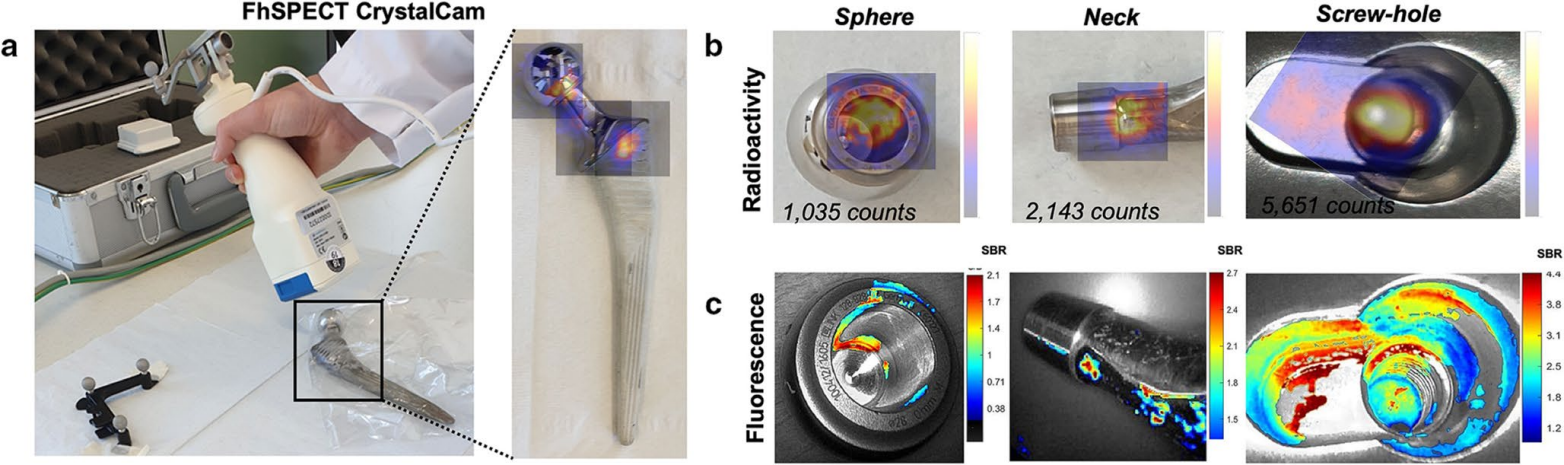
Visualization of bacterial infection using [^99m^Tc]Tc-UBI_29–41_-Cy5. Radioactivity imaging using a handheld gamma probe allowed **A** Assessment of the presence and location of the bacterial infection on the femur prosthesis and **B** Quantification of the level of bacterial infection based on imaging and gamma counting. **C** Fluorescence imaging of the corresponding locations of bacterial infection, with the bacterial load represented based on the color-coded signal-to-background (SBR) ratio. Adapted from [93]. Published with permission under a Creative Commons Attribution 4.0 International License. https://creativecommons.org/licenses/by/4.0/

Prosthetic joint infections remain a major clinical challenge, particularly due to the formation of bacterial biofilms on implant surfaces. These biofilms confer resistance to host immune clearance and antimicrobial therapy, often necessitating surgical intervention. Despite advances in diagnostic imaging, no specific clinical tracers are routinely available to identify bacteria within biofilms on infected prostheses [92]. In this respect, [^99m^Tc]Tc-UBI_29 − 41_-Cy5 (Fig. SI-1B) was evaluated in a follow-up study to detect bacteria in biofilm on infected explants of human femoral prostheses [93]. The rationale was to topically apply the tracer to evaluate imaging, quantify the bacterial load, and develop imaging-guided cleaning strategies for infected prostheses (Fig. 13A-C). [^99m^Tc]Tc-UBI_29 − 41_-Cy5 effectively stained bacteria in biofilms, allowing qualitative and quantitative monitoring of bacterial eradication over time. It allowed the visualization of the bacterial load on femoral implants using clinical-grade image guidance modalities. Combined real-time fluorescence and nuclear imaging were used to monitor the effects of prostheses cleaning. After cleaning with chlorhexidine, 28% to 44% of the radioactive and fluorescent signal remained, indicating that 500 to 2,000 viable bacteria were still present after the cleaning process. These findings warrant an alternative cleaning setup to prevent reinfections after DAIR. Such studies employing alternative cleaning procedures are now in progress, aiming to guide the cleaning of infected orthopaedic prostheses [93].

In this context, the visualization of periprosthetic infections differs significantly from that of aseptic loosening [3]. Thus, a generic, pathogen-targeting MMI tool, such as [^99m^Tc]Tc-UBI_29 − 41_-Cy5, represents a new diagnostic technology that can detect metabolically less active bacteria in biofilms on human explants [93].

## Utilizing UBI_29–41_ as a strategic platform for bacterial pretargeting studies

**Fig. 14:**
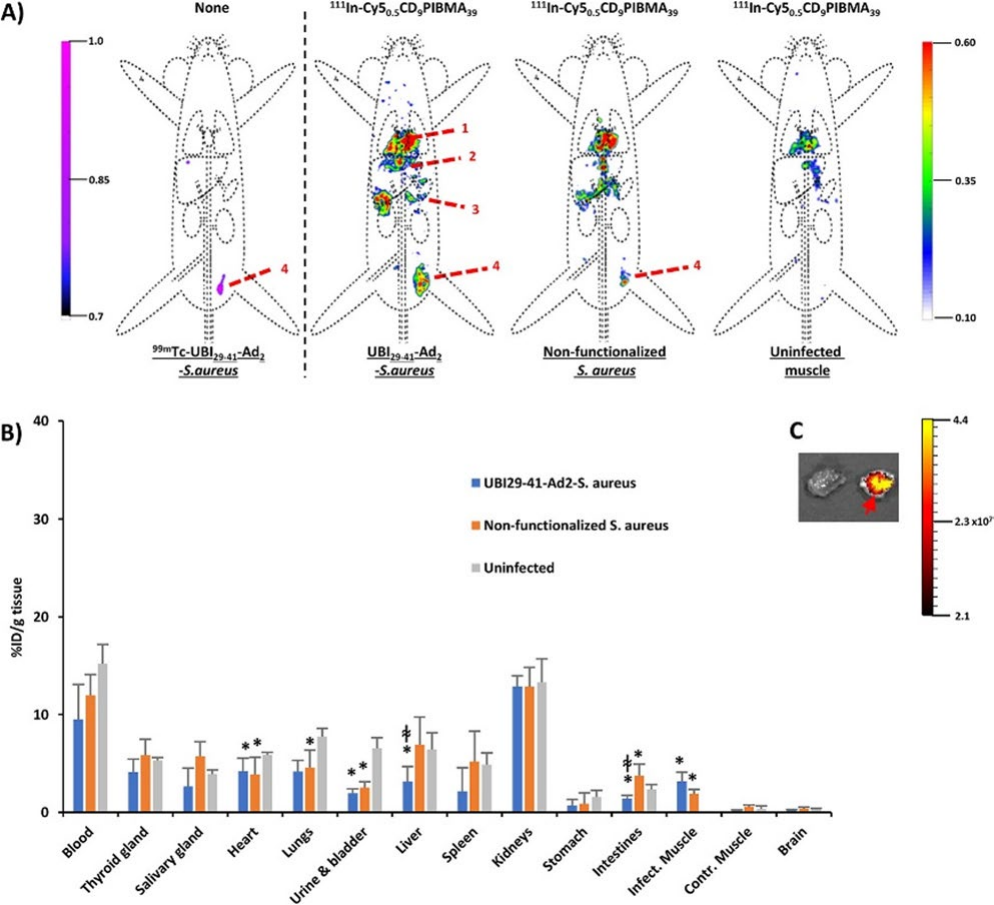
Cargo accumulation in bacteria-inoculated muscle. **A** In vivo SPECT imaging of [^99m^Tc]Tc-UBI_29 − 41_-Ad_2_-labelled *S. aureus* 44 h after inoculation in the thigh muscle (left panel; purple-to-pink colour coding) and imaging of host-vector [^111^In]In-Cy5_0.5_CD_9_PIBMA_39_ (rainbow colour coding). Clear colocalization of ^99m^Tc- and ^111^In-activity was seen for mice colonized with Ad-functionalized *S. aureus*. In contrast, colocalization of such signals was either less pronounced or absent in non-functionalized bacteria or uninfected muscle, respectively. Organs are marked as (1) heart/lungs, (2) liver, (3) intestines, and (4) inoculation site (thigh muscle). **B** Biodistribution studies of [^111^In]In-Cy5_0.5_CD_9_PIBMA_39_ in mice inoculated with [^99m^Tc]Tc-UBI_29 − 41_-Ad_2_-functionalized *S. aureus* (blue bars), non-functionalized *S. aureus* (orange bars), or no infection (gray bars). The data (mean ± SD ratios of the %ID/g) showed comparable activity in blood and major tissues for the 3 groups, whereas uptake between infected muscle and controls differed significantly (*n* = 6 for each group). * *p* < 0.05. **C** Ex vivo fluorescence imaging of muscle tissue corroborated the findings, with the infected muscle indicated with a red arrow. The scale bar indicates the intensity of fluorescence expressed as photons/s/cm^2^. Adapted from [94]. Published with permission, Copyright 2021 American Chemical Society

Next, the supramolecular host-guest approach for cyclodextrin (CD)/adamantane (Ad)-mediated targeting of inoculated bacteria in mice was introduced [94], where UBI_29 − 41_ facilitates scaffolds of [^111^In]In-Cy5_0.5_CD_9_PIBMA_39_ to bind to *S. aureus* bacteria via Ad functionalization introduced via pretargeting bacteria with UBI_29 − 41_-Ad_2_. For the bacteria tracking studies, inoculated *S. aureus* bacteria were tracked and traced either in a thigh muscle (Fig. 14) or the liver [94]. The bacteria used for inducing infections were functionalized with [^99m^Tc]Tc-UBI_29 − 41_-Ad_2_ (primary vector) to appoint and quantify the bacterial load, and to create an in vivo target for the secondary host-vector at 24 h post-inoculation.

The secondary vector for complexation utilized a [^111^In]In-Cy5_0.5_CD_9_PIBMA_39_ polymer, which was administered intravenously. Bacteria-specific delivery was evaluated using dual-isotope SPECT imaging and biodistribution studies, as well as fluorescence at 4 h post-injection (p.i.). Mice inoculated with non-functionalized *S. aureus* and mice without an infection served as controls. Dual-isotope SPECT imaging demonstrated that [^111^In]In-Cy5_0.5_CD_9_PIBMA_39_ colocalized with [^99m^Tc]Tc-UBI_29 − 41_-Ad_2_-labelled bacteria in both muscle and liver. In the inoculated muscle, a 2-fold higher uptake level (3.2 ± 1.0%ID/g) was noted compared to inoculations with non-functionalized bacteria (1.9 ± 0.4%ID/g), and a 16-fold higher uptake level compared to non-infected muscle (0.2 ± 0.1%ID/g). The hepatic accumulation of the host-vector was nearly 10-fold higher (27.1 ± 11.1%ID/g) compared to the non-infected control (2.7 ± 0.3%ID/g; *p* < 0.05). Fluorescence imaging of the secondary vector corroborated the findings from SPECT imaging and biodistribution. This study demonstrated that using two different bacterial models in soft tissue and liver, supramolecular host − guest complexation can be harnessed to achieve an in vivo cargo-delivery strategy. Expanding the supramolecular host-guest approach for cyclodextrin/adamantane-mediated targeting of inoculated bacteria in mice for vaccine development [95], UBI_29 − 41_ facilitates the scaffold Cy3_1.5_CD_100_PIBMA_389_ to bind to Gram-positive and Gram-negative bacteria, also using the Ad functionalization introduced via pretargeting bacteria with UBI_29 − 41_-Ad_2_.

The first line of defense is sentinel immune cells, such as macrophages, which phagocytose intact radiolabelled bacteria, thereby initiating immune responses. The bacterial surface composition is a crucial element that determines macrophage signalling. Pretargeting technology could be utilized to study the immune response by adjuvanting bacteria and the role of bacterial cell surface composition in immunological responses. Supramolecular host − guest chemistry between polymers with CD and UBI_29 − 41_-Ad_2_ was utilized to efficiently load versatile chemical scaffolds onto Gram-positive and Gram-negative bacteria [96]. This method provides a potential tool for investigations to alter the immunogenic properties of (bacterial) pathogens for either dissecting the complex host − pathogen interactions involved in infectious disease etiology or developing novel interventions against infectious diseases.

## Utilizing the MMI concept as a biomarker for measuring treatment response

**Fig. 15:**
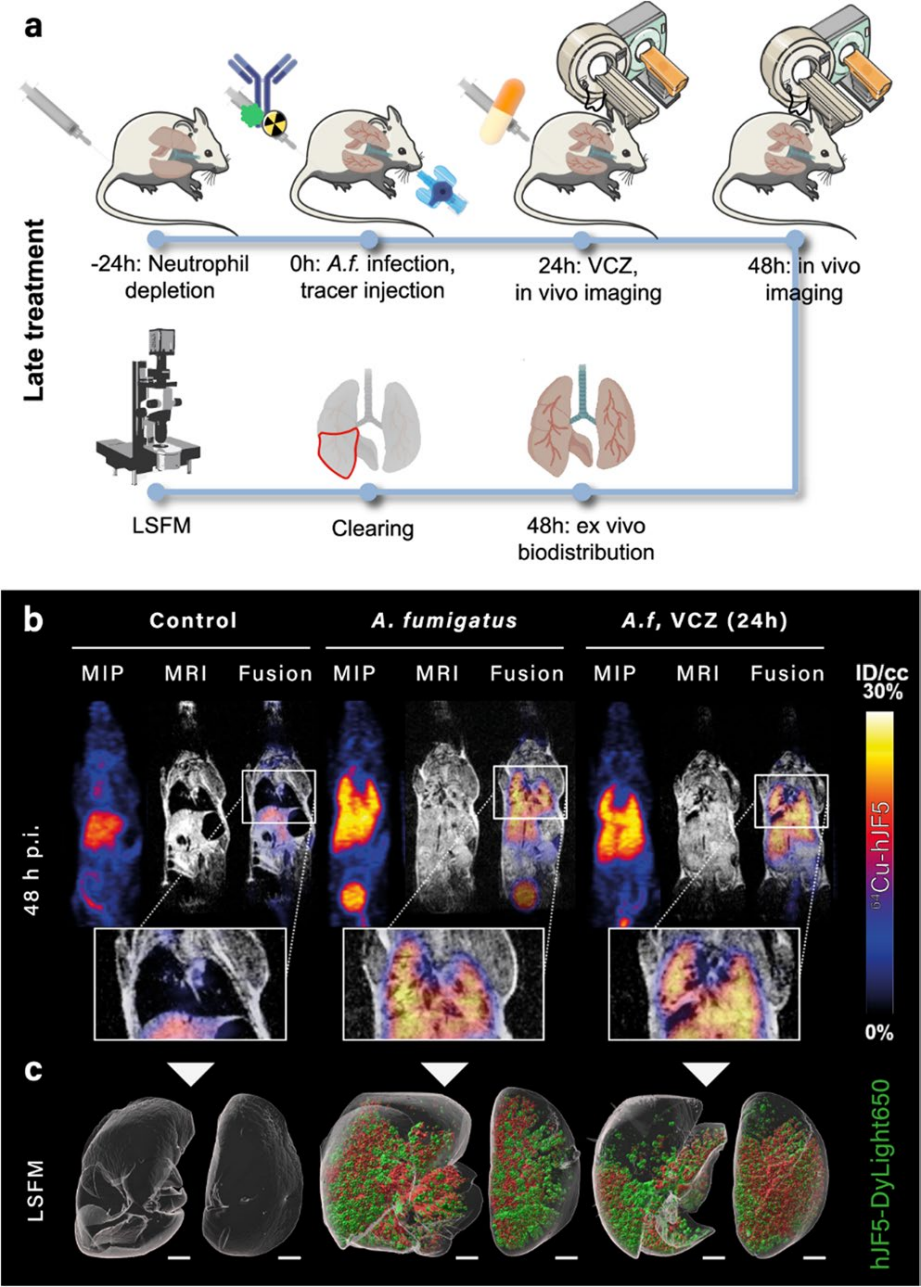
Schematic representation of the experimental design for investigation of [^64^Cu]Cu-hJF5-DyLight650 for imaging of *A. fumigatus* (A.f.) infection in uninfected (control), untreated, and 24 h post-inoculation voriconazole (VCZ) treated in A.f infected mice using immune-PET/MRI and light sheet fluorescence microscopy (LSFM). Adapted from [64]. Published with permission under Creative Commons Attribution 4.0 International License.https://creativecommons.org/licenses/by/4.0/

Monitoring treatment response is a critical priority in nuclear molecular imaging of infections. Despite the development of numerous target-specific radiotracers for infection diagnostics, the ability to assess therapeutic efficacy in vivo remains an unmet clinical need [97]. To achieve full translational potential, infection-targeting radiotracers must fulfil both diagnostic and treatment response criteria. For example, the ^64^Cu-labelled *Aspergillus*-specific monoclonal antibody (hJF5), [^64^Cu]Cu-hJF5, was investigated for assessing treatment response in neutropenic mice with induced invasive pulmonary aspergillosis after treatment with the antifungal agent voriconazole [64]. Despite the accuracy of the radiotracer in diagnosing invasive pulmonary aspergillosis, the distribution of the radiotracer signal was the same for both treated and untreated cohorts. For better understanding, the study revealed a correlation between signal quantification and infection burden in relation to treatment response using the hybrid labelled [^64^Cu]Cu-hJF5-DyLight_650_ setting (excitation/emission: 652/672nm) (Fig. 15). For this study, animal lung perfusion was performed to minimize the non-specific binding attributed to the extended blood circulation time of the hybrid agent, which might hinder clinical translation for application in treatment response monitoring [64].

The study further highlights the challenges and important parameters involved in establishing a treatment monitoring protocol for infection imaging. The study also demonstrates that PET/SPECT using [^64^Cu]Cu-hJF5 alone might be limited in accurately assessing treatment response, especially for radiotracers targeting areas that show background signals due to non-specific distribution. Therefore, a threshold signal should be established using morphological techniques, such as CT or MRI, to guide response assessment at different radiotracer accumulations. In addition, the pharmacodynamics and pharmacokinetics of the treating agent may influence the tracer distribution; therefore, this should be considered when setting up appropriate time points for imaging post-treatment studies.

## Critical discussion

Recent advances in nuclear imaging of infection have introduced novel tracers that directly target pathogens, offering improved specificity over traditional inflammation-based methods. They are shifting focus from host immune responses to pathogen-specific detection due to leveraging small molecules with a well-know mechanism of action [8]. While conventional tracers like [¹⁸F]FDG and labeled leukocytes primarily highlight inflammation, new tracers are being developed to bind directly to microbial components, such as bacterial cell walls or metabolic pathways [98, 99]. These innovations enhance diagnostic accuracy by distinguishing infection from sterile inflammation and allow for better monitoring of antibiotic delivery and therapeutic response [98]. Whilst we herein highlight currently available MMI strategies - with several novel nuclear and non-nuclear imaging techniques arising -the platform for other suitable MMI agents is concurrently opening for highly relevant research and innovations. As highlighted by Polvoy et al. nuclear imaging techniques offer unique advantages in visualizing infectious pathophysiology beyond structural changes, and integrating these with optical modalities may bridge the gap between molecular insight and surgical precision [99].

### Aligning multimodal imaging with clinical Decision-Making

The true value of MMI lies not in technical novelty alone, but in its capacity to inform and enhance clinical decision-making across disciplines. To realize this potential, preclinical workflows must evolve from isolated proof-of-concept studies into integrated platforms that reflect the complexity, urgency, and diversity of real-world infectious disease management [100]. MMI agents, particularly hybrid tracers that combine nuclear and optical modalities, should be designed not only for diagnostic accuracy but also for operational utility. The multi-modality includes enabling real-time visualization during surgery, guiding targeted debridement, and supporting longitudinal monitoring of therapeutic response. Such functionality is especially critical in settings where conventional imaging fails to distinguish viable infection from sterile inflammation, or where anatomical imaging lacks molecular specificity.

From a translational standpoint, multimodal molecular imaging (MMI) agents must be compatible with clinical infrastructure to ensure seamless integration into diagnostic and surgical workflows. These workflows include: *i*) fluorophores matched to the excitation/emission profiles of surgical imaging systems; *ii*) radiolabels that are chemically stable, safe for human use, and aligned with regulatory pathways; and *iii*) pharmacokinetic profiles that support both preoperative planning and intraoperative visualization. Beyond technical compatibility, integrating imaging with antimicrobial stewardship, such as identifying non-responders, confirming infection resolution, or detecting pathogen dispersion, can reduce overtreatment, guide targeted therapy, and improve patient outcomes. As emphasized by Messacar et al. diagnostic and antimicrobial stewardship are essential to translating molecular diagnostics into actionable clinical decisions, ensuring that advanced imaging technologies contribute meaningfully to infection management [101]. To transition MMI from a research tool to a clinical asset, agents must be validated in infection models that reflect human pathology, with standardized imaging protocols and tracer designs that balance specificity, stability, and clearance. Addressing these translational challenges will position MMI as a keystone of precision infectious disease management, bridging molecular insight with actionable care. It has been previously noted that the clinical adoption of MMI will benefit not only from its innovation alone, but also from its integration into therapeutic decision-making through robust validation and harmonized workflows [97].

### Critical gaps in translating Infection-Specific MMI agents from bench to bedside

Despite significant advances in the development of multimodal imaging tracers for infectious diseases, preclinical studies often fail to address the full spectrum of clinical needs, particularly those extending beyond the domain of nuclear medicine. Recently, Auletta et al. highlighted the emerging radiopharmaceuticals for bacterial imaging, even though some bias related to animal selection and index test limited their translational success: the lack of standardized infection models and experimental settings has (so far) limited the possibility of comparing studies and the translation to more extensive human studies [8, 102] Herein we provide a summary of limitations (technical and strategic) including respective mitigations to improve the development of MMI agents of infection (Table 1).

**Table 1:** General study limitations for the preclinical development of tracers for multimodal imaging of infection

Category	Problem/Limitation	Mitigation Strategy
Study Design	Artificial infection models (e.g., intramuscular inoculation)	Use clinically relevant models (e.g., biofilm, implant-associated, or chronic infection models)
	Small sample sizes and lack of longitudinal imaging	Incorporate time-course imaging and power calculations for statistical robustness
	Single-pathogen species models	Include poly-microbial or opportunistic pathogens to reflect on existing clinical diversity
	Lack of standardized imaging protocols	Adopt harmonized imaging workflows and quantitative metrics (e.g., SUV, TBR)
Translational Impact	Limited validation in large animals or human tissues	Extend studies to porcine or non-human primate models; use ex vivo human explants
	Non-specific targeting (e.g., charge-based or inflammation markers)	Develop tracers against pathogen-specific antigens or virulence factors
	No assessment of tracer performance during therapy	Include antibiotic or surgical intervention arms to evaluate clinical utility
Methodological Oversights	Inadequate controls (e.g., sterile inflammation)	Include matched controls for inflammation, necrosis, and non-infectious tissue damage
	Co-injection of separate radio/fluorescent tracers instead of true hybrids	Engineer single-molecule dual-labelled tracers with matched pharmacokinetics
	Lack of biodistribution and metabolism data	Perform full organ biodistribution and metabolite analysis using gamma counting and LC-MS
Compound-Specific Flaws	Poor pharmacokinetics or high off-target uptake	Optimize linker chemistry, molecular weight, and charge distribution
	Fluorophore-induced changes in tracer behaviour	Validate tracer performance with and without fluorophore; use minimally perturbing dyes
	Enzymatic degradation (e.g., maltodextrins)	Modify terminal sugar residues or use enzyme-resistant analog
	Long circulatory half-life of full-size antibodies	Focus on antibody fragments, affibodies, or nanobodies for faster clearance and reduced background (noise) signal
	Use of non-clinically approved dyes or chelators	Select fluorophores and chelators with known safety profiles or clinical precedent

While many agents demonstrate promising uptake and specificity in controlled laboratory settings, their translational relevance remains constrained by limitations in study design, compound behaviour, and methodological rigor. A critical appraisal of these pitfalls reveals opportunities for refinement and alignment with real-world clinical demands, including surgical guidance, antimicrobial stewardship, and diagnostic precision. To meet the broader clinical demands of infection management, imaging agents must evolve from static diagnostic tools to dynamic decision-support platforms. In surgical settings, fluorescent tracers should enable real-time visualization of infected tissue, facilitating precise debridement and thereby reducing the likelihood of recurrence. In antimicrobial stewardship, imaging can help distinguish between active infection and post-treatment inflammation, thereby minimizing unnecessary antibiotic use. For treatment monitoring, longitudinal imaging is crucial for tracking therapeutic response, particularly in immunocompromised or critically ill patients. Ultimately, compatibility with portable imaging systems is crucial for seamless integration into operating rooms, intensive care units, and outpatient clinics. MMI is considered most impactful when it enables cross-validation of infection localization, supports real-time surgical guidance, and informs treatment response. To achieve this, preclinical workflows must be tightly integrated, from tracer design to imaging execution and data analysis. More emphasis must be placed on addressing clinical relevance, rather than just technical success.

### Practical considerations for optimizing MMI workflows in preclinical infection studies

**Fig. 16:**
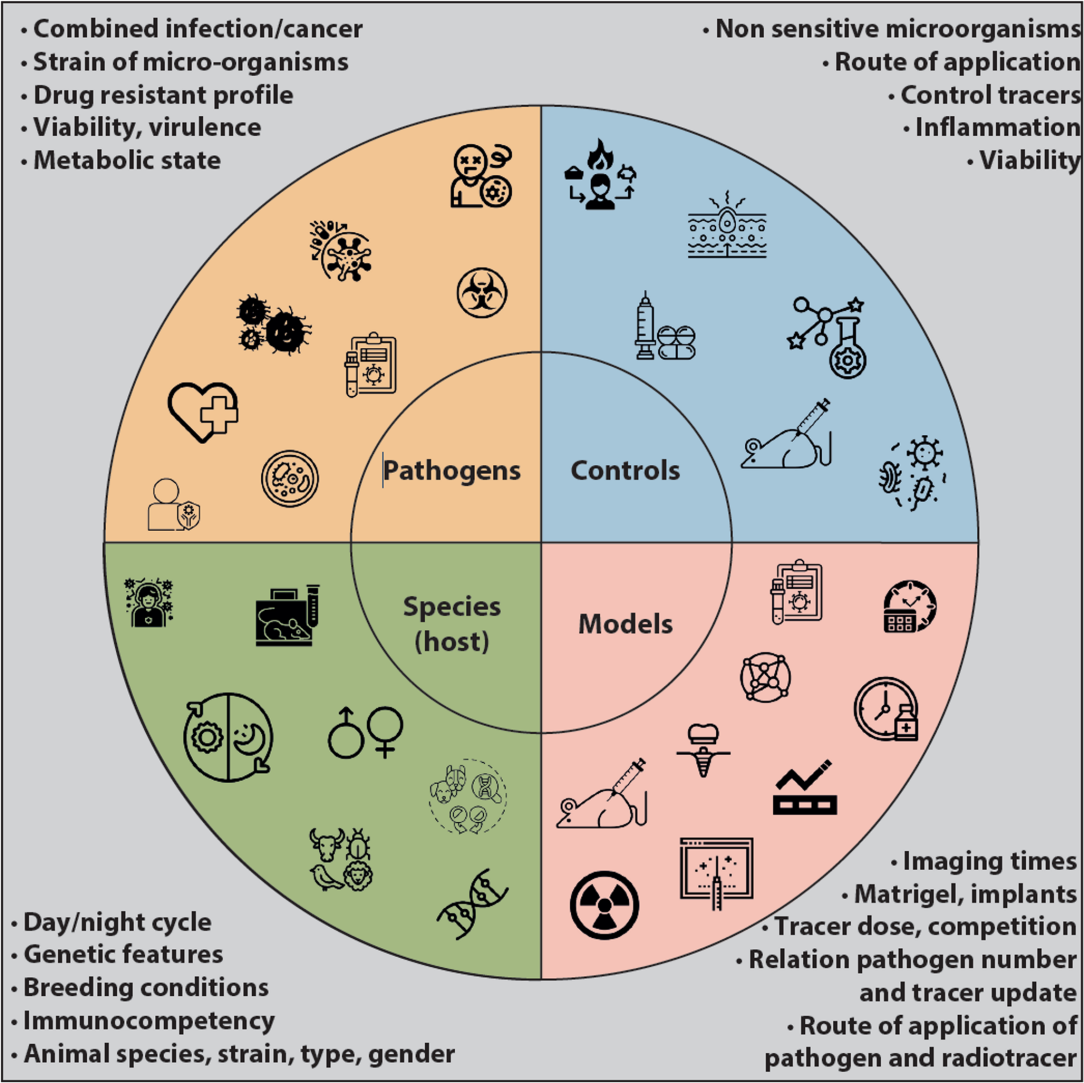
Schematic representation of the most relevant parameters to consider when planning new experiments in animal models targeting pathogens. Image created using icons from the Noun Project

To fully harness the potential of MMI in preclinical research, particularly for infection imaging, workflows must be meticulously integrated across imaging modalities. When designing animal studies that target pathogens in vivo, a clear understanding of key criteria, experimental parameters, and available resources is essential to ensure optimal study design, workflow efficiency, and data quality. An overview of the most relevant parameters for consideration before commencement of MMI animal studies of infection is represented in Fig. 16.

Based on the latter inputs, the following practical considerations may serve as a blueprint for ensuring technical rigor, data integrity, and translational relevance:

1. *Establish Cross-Modality Experimental Planning* - Initiate study design with coordinated input from experts in nuclear, optical, and computational imaging. Address critical variables, such as early sample preparation, fiducial placement, probe stability, inter-instrument transfer, and modality-specific controls, to ensure seamless execution and meaningful data integration.
2. *Sequence Imaging Modalities Strategically* - When applying multiple modalities to the same sample, carefully determine the acquisition order to preserve sample integrity. Consider risks such as photobleaching, thermal degradation, or cellular compromise, and prioritize modalities based on their invasiveness and sensitivity to these risks.
3. *Define Analytical Endpoints with Computational Alignment* - Engage image analysis specialists to align acquisition parameters with downstream processing needs. Clearly articulate biological endpoints and leverage community resources (e.g., forum.Image.sc) to identify or adapt existing tools for multimodal data fusion and quantification.
4. *Coordinate Instrument Access and Sample Logistics* - If imaging platforms are distributed across facilities, establish clear protocols for sample transit, timing, and storage. Facilitate communication between technical teams to synchronize workflows and consider leveraging integrated infrastructures such as Euro-BioImaging for streamlined access to multimodal instrumentation.
5. *Implement Robust Data Management Frameworks* - Before acquisition begins, define standardized practices for data storage, nomenclature, and format compatibility. Ensure that all imaging protocols and metadata are captured comprehensively to support reproducibility, cross-modality correlation, and potentially consider open-access dissemination.
6. *Pilot and Refine Emerging Workflows* - For novel imaging strategies, first conduct small-scale pilot studies to identify potential interference between modalities and optimize acquisition parameters. Iterative refinement at this stage minimizes downstream complications and enhances overall workflow robustness.

Overall, conducting small-scale pilot experiments serves as a strategic precaution in hybrid infection imaging. It enables the testing and refinement of expected workflows before full-scale implementation, ensuring that imaging modalities do not interfere with one another and that data acquisition is both reliable and interpretable. This iterative approach can mitigate technical risks, address cross-modality matching, and help to achieve reproducible, clinically relevant imaging outcomes.

## Perspective and outlook

Apart from each other, nuclear imaging and optical imaging both contribute to the imaging and diagnostics of bacteria in infections, depending on the specific interaction of the diagnostic agent with the pathogens and host cells or metabolic processes. The hybrid moiety, which involves adding a fluorescent dye to a clinically validated nuclear medicine tracer, could expand existing diagnostic applications into intraoperative utility through multiplexing and significantly change how interoperative infection surgery is conducted [103]. MMI agents are capable of *i*) whole-body or local nuclear imaging for diagnosis and surgical planning and *ii*) e.g., NIRF imaging for image-guided surgical interventions. As already evident in oncology, combining diagnostic SPECT (or PET) imaging capabilities with image-guided surgery using a single imaging agent could significantly improve patient outcomes [104, 105]. Moreover, adding an NIRF dye to a radiotracer enables the definitive correlation between the diagnostic imaging used for surgical planning and image-guided surgery. Besides their clinical utility, hybrid-labelled imaging agents provide a quantitative method to validate NIRF tomography using SPECT or PET values, expressed as the percentage of the injected dose per gram of tissue (%ID/g). At the same time, imaging infections after the co-administration of a singly labelled nuclear and a single labelled fluorescent agent containing the same targeting moiety may seem like an uncomplicated strategy. However, differences in pharmacokinetics, binding affinities, physical half-life, and the complexities of co-administration between the two distinct imaging agents restrict a practical application. Studies evaluating the biodistribution and pharmacokinetic properties of single-labelled nuclear and fluorescent agents have yielded conflicting results. Specifically, targeting moieties labelled with different chelators and radioisotopes exhibit varying biodistribution patterns and PK-values, which can impact imaging performance [106, 107].

In a potential clinical setting, patients with potentially resistant bacteria in a severe, deep-seated infection, or, for example, a fever of unknown origin, could receive an injection of an infection-specific MMI agent and undergo a standard nuclear imaging workflow that accurately localizes infected tissues or foci. Following a predetermined decay period, depending on the physical half-life of the radioisotope (i.e., 24 h post-injection), patients would then proceed to the operating theatre, where surgeons may analyse whole-body, tomographic nuclear images to aid in determining their surgical plan. Before resection, in open or (robotic) laparoscopic settings, imaging with intraoperative NIRF modalities is available to confirm sites with optically enhanced tissue uptake. The optical imaging modality identifies potential infected regions and margins in real-time, enabling surgeons to perform more accurate debulking and cleaning procedures. Immediately after the intervention procedure, NIRF imaging would be capable of assessing the effectiveness of the intervention and determining if additional surgery is warranted to remove remaining tissues that still exhibit a fluorescent signature. Radioactive counting, histological evaluation, and microbiological culturing of resected tissues would then confirm the precise molecular targeting of the tracer and the presence of the pathogens. In such cases, additional or alternative antibiotic treatment can be applied to reduce the likelihood of reinfection.

Even an introduction of tri-modal (hybrid) imaging has been under investigation; such an imaging agent capable of MRI, SPECT, and fluorescence imaging was generated after labelling superparamagnetic iron oxide nanoparticles (SPIONs) with ^99m^Tc and an Alexa-fluorophore dye [108]. The results demonstrate that a single imaging agent can be utilized for MMI, and a single hybrid contrast agent enables simultaneous hybrid imaging, allowing for single-modality imaging at different time points. The hybrid nanoprobe demonstrated specific accumulation in sentinel lymph nodes; however, bacterial imaging was not performed. The advantage is that this tracer allows disease detection and personalized treatments. The weakness was the low contrast between the lymph nodes and surrounding tissue without SPIONs, and the non-homogeneous uptake of nanoparticles within the sentinel lymph node.

Looking further ahead, the integration of novel MMI agents into clinical workflows offers a compelling opportunity to redefine how complex infections are diagnosed, localized, and treated with precision medicine. The convergence of nuclear and optical modalities within a single tracer may soon enable a seamless transition from whole-body diagnostic imaging to intraoperative precision, thereby bridging preoperative planning with real-time surgical decision-making. To fully realize this potential, future efforts must prioritize chemically coherent tracer design, matched pharmacokinetics, and standardized acquisition protocols that support both imaging depth and surgical usability. As hybrid agents evolve to incorporate pathogen-specific targeting and regulatory-ready components, their role in managing complex infections, such as deep-seated abscesses, implant-associated biofilms, or fever of unknown origin, will expand beyond diagnostics into therapeutic guidance. Ultimately, the clinical adoption of MMI agents will depend not only on technical innovation but on their ability to integrate across disciplines, streamline workflows, and deliver actionable insights at every stage of infection care.

## Summarizing statements

In this review, we highlight a range of promising hybrid tracers designed for multimodal molecular imaging (MMI) of infections. While several constructs demonstrate compelling preclinical performance, none have yet transitioned into routine clinical use. Accelerating this translation will require focused development of select hybrid agents that combine the diagnostic depth of nuclear imaging with the intraoperative precision of optical modalities, i.e., a “best-of-both-worlds” strategy for clinical translation would speed up this process [8, 109], which was already shown with clinical promise in oncology [105, 110]. To support this transition, it is essential to rigorously define preclinical parameters, including the selection of infection models, tracer pharmacokinetics, and modality-specific controls. Studies have shown that co-administered nuclear and fluorescent agents, even when sharing the same targeting moiety, can exhibit divergent biodistribution and clearance profiles due to differences in chelator chemistry and isotope half-life [111, 112]. These discrepancies underscore the need for chemically coherent hybrid agents with matched kinetics and validated imaging performance. Looking forward, the clinical integration of MMI agents offers a transformative opportunity to improve infection care; thus, MMI agents can evolve into actionable platforms for precision infectious disease management.

## Supplementary Information

Below is the link to the electronic supplementary material.

ESM 1 PPTX (336 KB)

## Data Availability

The datasets generated during and/or analysed during the current study are available from the corresponding author on reasonable request.
