# Supplementary material for: Recent advances in multimodal imaging of infections: research highlights using Nuclear-Optical imaging
Source: Eur J Nucl Med Mol Imaging. 2026 Jan 10;53(5):2903–26. doi: 10.1007/s00259-025-07724-y (PMC13013333; doi:10.1007/s00259-025-07724-y)
Supplement: Supplementary file 1 — PPTX (336 KB) [file 259_2025_7724_MOESM1_ESM.pptx]

## Slide 1
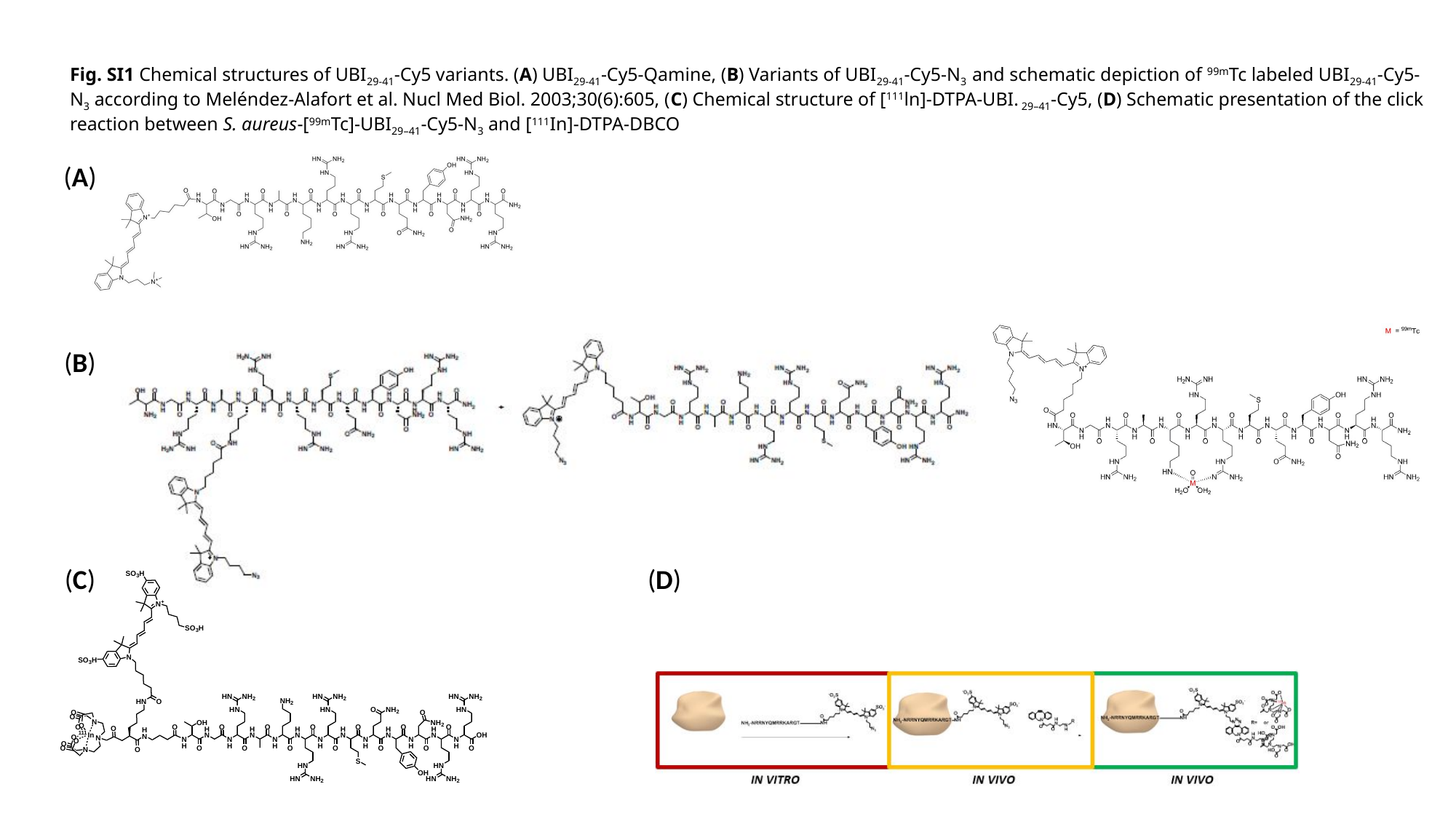

Fig. SI1 Chemical structures of UBI29-41-Cy5 variants. (A) UBI29-41-Cy5-Qamine, (B) Variants of UBI29-41-Cy5-N3 and schematic depiction of 99mTc labeled UBI29-41-Cy5-N3 according to Meléndez-Alafort et al. Nucl Med Biol. 2003;30(6):605, (C) Chemical structure of [111ln]-DTPA-UBI. 29–41-Cy5, (D) Schematic presentation of the click reaction between S. aureus-[99mTc]-UBI29–41-Cy5-N3 and [111In]-DTPA-DBCO
(A)
(B)
(C)
(D)
